# Association between *CYP2E1* polymorphisms and colorectal cancer risk: a systematic review and meta-analysis

**DOI:** 10.1038/s41598-022-24398-w

**Published:** 2022-11-23

**Authors:** Mohamad Ayub Khan Sharzehan, Hilary Sito, Noraidatulakma Abdullah, Athanasios Alexiou, Marios Papadakis, Rahman Jamal, Shing Cheng Tan

**Affiliations:** 1 UKM Medical Molecular Biology Institute, Universiti Kebangsaan Malaysia, Cheras, Kuala Lumpur, Malaysia; 2 Department of Science and Engineering, Novel Global Community Educational Foundation, Hebersham, Australia; 3 AFNP Med Austria, Wien, Austria; 4 Department of Surgery II, University Hospital Witten-Herdecke, University of Witten-Herdecke, Heusnerstrasse 40, 42283 Wuppertal, Germany

**Keywords:** Cancer, Genetics, Biomarkers, Medical research, Molecular medicine

## Abstract

*CYP2E1* encodes an enzyme that participates in the activation of several carcinogenic substances. Thus, numerous studies have investigated the association between *CYP2E1* polymorphisms and colorectal cancer (CRC) risk, but inconclusive results have been obtained. We performed a meta-analysis to precisely evaluate the relationship of *CYP2E1* rs2031920, rs3813867, and rs6413432 polymorphisms with the susceptibility to CRC. Scopus, Web of Science and PubMed databases were searched to identify eligible studies, and the association between the polymorphisms and CRC risk was then quantitatively synthesized using different genetic models. Eighteen studies with 23,598 subjects were selected for inclusion into the analysis. Significant association between rs2031920 and an increased CRC risk was observed in homozygous (OR = 1.496, 95% CI 1.177–1.901, *P* = 0.001), recessive (OR = 1.467, 95% CI  1.160–1.857, *P* = 0.001) and allele (OR = 1.162, 95% CI  1.001–1.349, *P* = 0.048) models. Significant association was not found for rs3813867 and rs6413432 (*P* > 0.05). In conclusion, our results suggest that rs2031920, but not rs3813867 and rs6413432, is associated with the risk of CRC.

## Introduction

Colorectal cancer (CRC) is one of the most common cancers worldwide and is associated with significant morbidity and mortality^1^. In 2020 alone, nearly two million new CRC cases and one million CRC-related deaths were reported^2^. Age is a well-established risk factor for CRC along with other environmental risk factors such as physical inactivity, obesity, high intake of red, low intake of fiber, tobacco smoking, and alcohol consumption^3,4^. In addition, genetics has unequivocally been implicated as a key determinant in the development of CRC^5^. Polymorphisms in cancer-related genes may therefore influence interindividual susceptibility to CRC^6^.

*CYP2E1* encodes cytochrome P450 2E1 (*CYP2E1*), a phase I enzyme involved in the process of xenobiotic metabolism. *CYP2E1* plays a key role in the conversion of xenobiotics into several highly reactive intermediate metabolites prior to their elimination by phase II enzymes^7^. For example, it is known to activate low-molecular-weight procarcinogens such as nitrosamines into active carcinogens directly involved in digestive tract oncogenesis. Consequently, higher *CYP2E1* activity has been associated with higher rates of cancer progression^8^. The *CYP2E1* gene contains more than ten well-characterized single nucleotide polymorphisms (SNPs) that may influence the activity of the enzyme and thus cancer risk^9^. The most commonly studied polymorphisms in CRC include rs3813867 (conventionally known as *PstI*) and rs2031920 (conventionally known as *RsaI*) in the 5’-flanking regions of the gene, as well as rs6413432 (conventionally known as *DraI*) in intron 6. These polymorphisms are known to have functional effects on cells. In particular, rs2031920 and rs3813867 have been associated with increased transcriptional and enzymatic activity of the gene. In addition, increased transcriptional activity of the *CYP2E1* gene has been discovered in association with rs6413432, which is also associated with DNA single-strand breaks known to lead to cancer^10^. This is one of the reasons why we focused on these three polymorphisms in this study.

In addition, although these polymorphisms are frequently studied, the association between these polymorphisms and the risk of CRC remains inconclusive. For example, a study in the Hungarian population showed that the variant allele of rs3813867 was positively associated with an increased risk of CRC^11^. However, there were also studies showing a lack of association between the same polymorphisms and the risk of CRC^8,12,13^. Likewise, for rs2031920, while Silva et al*.*^14^ showed that individuals carrying the variant allele had an increased risk of developing CRC, Kury et al*.*^15^ found no association between the polymorphism and the risk of CRC. Similar discrepancies were also noted for rs6413432^16–19^. These inconsistencies between the studies may be attributed to the small sample size of each study and the different genetic backgrounds and lifestyles of study participants from different populations. Meta-analysis can be used to resolve these inconsistencies. However, the last meta-analyses focusing on these three polymorphisms was published almost 9 years ago^20,21^ and new studies in this area have recently been added, which may lead to a different study result. This is another reason why we focus on these three polymorphisms. Therefore, in this work, a meta-analysis was conducted to obtain a more precise estimate of the association between *CYP2E1* rs3813867, rs2031920, and rs6413432 polymorphisms and CRC risk.

## Results

### Study selection and characteristics

**Figure 1:**
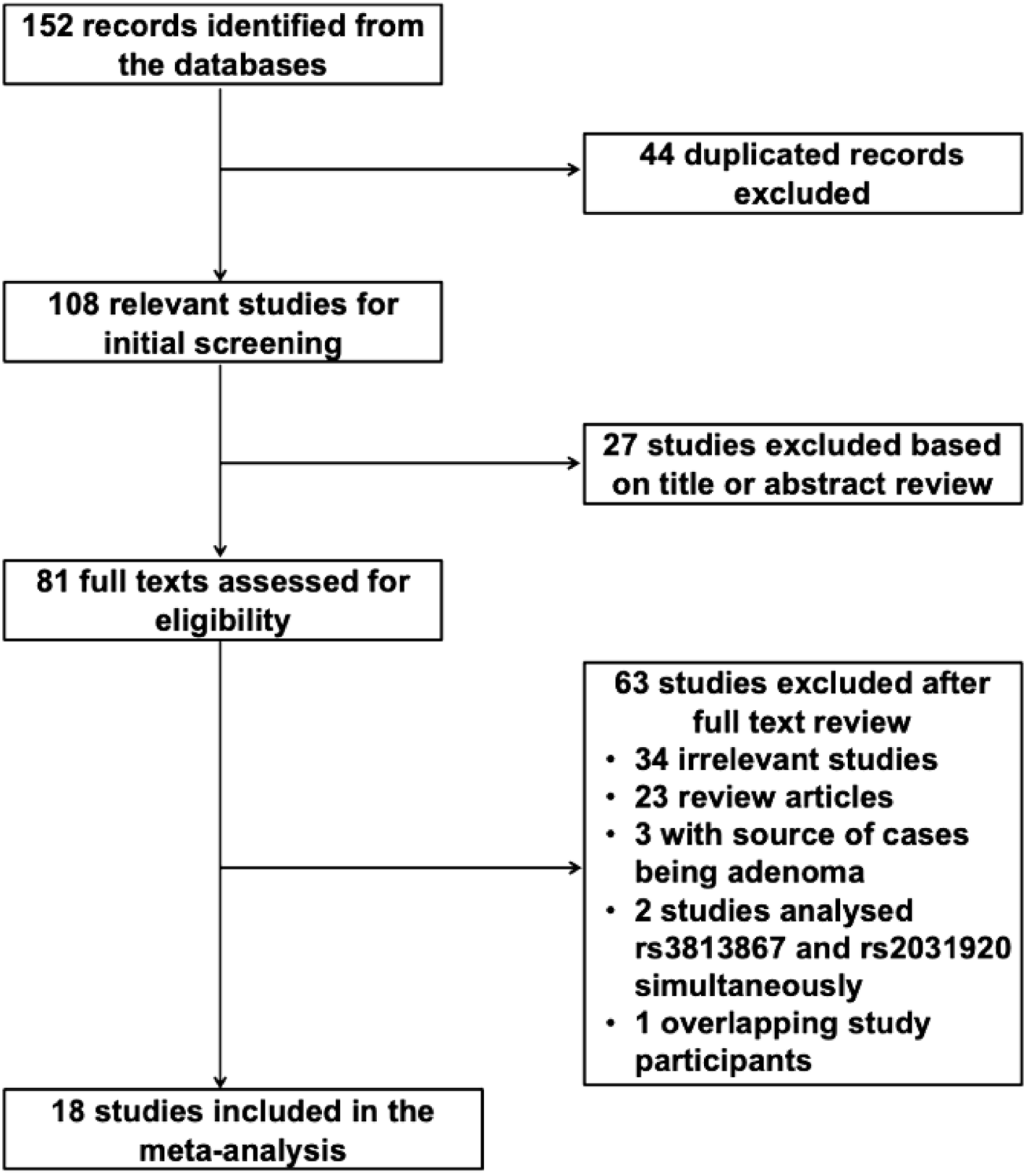
Flow diagram of search process.

The flow diagram of the study selection process is shown in Fig. 1. A total of 152 records were identified in the PubMed, Scopus and Web of Science databases. Of these, 44 records were identified as duplicates and removed. After screening the titles and abstracts of the remaining studies, 81 studies were identified as potentially relevant. When the full-texts were reviewed, 63 studies were excluded for the following reasons: (i) they reported data on other *CYP2E1* polymorphisms, were case-only studies, or did not contain useful information (N = 34); (ii) they were review articles (N = 23); (iii) the cases included patients with benign tumors such as adenomas (N = 3); (iv) they did not contain individual data for the rs3813867 and rs2031920 polymorphisms (N = 2); (v) study participants overlapped in more than one study (N = 1). Finally, 18 studies comprising a total of 10,302 cases and 13,296 controls were included in this meta-analysis.

The characteristics of the included studies are shown in Table 1. Of the included studies, two were in Chinese^22,23^ and the rest were in English. Seven studies were conducted on Asians^17,22–27^, seven studies were conducted on Caucasians^11,13–16,28,29^, and the remaining four studies included participants of other descendants such as Brazilians, Saudi Arabians or mixed ethnicities^8,18,19,30^. The most commonly used method for genotyping the polymorphisms was polymerase chain reaction-restriction fragment length polymorphism (PCR–RFLP), while a few studies used Taqman or microarray-based approaches. For the rs6413432 polymorphism, the distribution of genotypes in controls was consistent with HWE in all the included studies. For rs2031920, however, the genotype distribution deviated significantly from HWE in three studies^22,27,30^. For rs3813867, only one study reported a significant deviation from the HWE^22^. All of the studies had high methodological quality (≥ 5 stars on the Newcastle–Ottawa Scale) except for two studies^22,30^. The star ratings of the included studies are shown in Table 2.

**Table 1:** Characteristics of studies included in the meta-analysis.

Study	Year	Country	Ethnicity	Diagnosis of CRC	Genotyping method	Total subjects (case/control)	Genotype (case/control)			Power	HWE (*p* value)
							Wild type	Heterozygous	Variant		
**rs2031920**											
Chen^22^	2005	China	Asian	Diagnosis based on standard clinical criteria	PCR–RFLP	138/339	59/164	68/156	11/19	0.43	0.019
Chen^23^	2007	China	Asian	Histopathologically confirmed CRC	PCR–RFLP	313/433	185/266	106/154	22/13	0.41	0.095
Chong^17^	2014	Malaysia	Asian	Histopathologically confirmed CRC	PCR	175/520	106/359	60/143	9/18	0.94	0.424
Gao^24^	2007	China	Asian	Histopathologically diagnosed with primary CRC	PCR–RFLP	313/433	185/266	106/154	22/13	0.41	0.095
Kury^15^	2007	French	Caucasian	Personal history of CRC confirmed based on endoscopy and histology reports	Taqman	1013/1118	940/1027	67/90	6/1	0.08	0.498
Landi^13^	2005	Spain	Caucasian	Confirmed diagnosis of colorectal adenocarcinoma	APEX	320/261	305/251	15/8	0/2	0.05	0.000
Le Marchand^30^	2002	Mixed	Other	Confirmed diagnosis of primary adenocarcinoma	PCR	521/639	384/449	116/164	21/26	0.29	0.029
Morita^26^	2009	Japan	Asian	Histologically confirmed colorectal adenocarcinomas	PCR–RFLP	685/778	412/455	237/279	36/44	0.12	0.886
Sameer^27^	2011	India	Asian	Diagnosis based on surgery and biopsy reports	PCR–RFLP	86/160	46/112	15/20	25/28	0.99	0.000
Silva^14^	2012	Brazil	Caucasian	Histopathologically diagnosed with colorectal adenocarcinoma	PCR–RFLP	131/206	110/186	18/19	3/1	0.86	0.503
**rs3813867**											
Chen^22^	2005	China	Asian	Diagnosis based on standard clinical criteria	PCR–RFLP	139/338	79/209	56/121	4/8	0.33	0.047
Cotterchio^16^	2008	Canada	Caucasian	Pathology-confirmed (*International Classification of Diseases*, Ninth Revision)	Taqman	832/1247	784/1162	48/85	0/0	0.32	0.212
Fernandes^18^	2016	Brazil	Other	Clinical and histopathological diagnosis of sporadic CRC	PCR–RFLP	227/400	157/351	67/49	3/0	0.99	0.191
Kim^25^	2019	Korea	Asian	Pathology confirmed of cancer site locations	Taqman	971/658	631/411	294/212	46/35	0.25	0.267
Kiss^11^	2007	Hungary	Caucasian	Histologically confirmed diagnosis	PCR–RFLP	500/500	428/456	65/42	7/2	0.99	0.337
Kury^15^	2007	French	Caucasian	Personal history of CRC confirmed based on endoscopy and histology reports	Taqman	1013/1118	944/1029	67/88	2/1	0.22	0.529
Landi^13^	2005	Spain	Caucasian	Confirmed diagnosis of colorectal adenocarcinoma	APEX	299/341	283/323	15/17	1/1	0.05	0.141
Proenca^8^	2015	Brazil	Other	Clinical histopathological confirmed diagnosed with sporadic CRC	PCR–RFLP	74/199	66/171	8/28	0/0	0.24	0.285
**rs6413432**											
Chong^17^	2014	Malaysia	Asian	Histopathologically confirmed CRC	PCR	175/520	111/320	55/166	9/34	0.19	0.053
Cleary^28^	2010	Canada	Caucasian	Pathology-confirmed (*International Classification of Diseases*, Ninth Revision)	Taqman	1165/1291	925/1032	226/246	14/13	0.08	0.695
Cotterchio^16^	2008	Canada	Caucasian	Pathology-confirmed (*International Classification of Diseases*, Ninth Revision)	Taqman	834/1248	665/1008	161/228	8/12	0.13	0.822
Darazy^29^	2011	Lebanon	Caucasian	Histologically confirmed by specialists	PCR–RFLP	57/70	55/66	2/4	0/0	0.14	0.805
Fernandes^18^	2016	Brazil	Other	Clinical and histopathological diagnosis of sporadic CRC	PCR–RFLP	227/400	126/314	93/82	8/4	0.99	0.594
Saeed^19^	2013	Saudi Arabia	Other	Diagnosis based on standard clinical, endoscopic, radiological, and histological criteria	PCR–RFLP	94/79	66/51	23/28	5/0	0.05	0.055

**Table 2:** Quality assessment of the included studies.

Study	Selection				Comparability	Exposure			Total star
	Criteria				Criteria	Criteria			
	1	2	3	4	1	1	2	3	
Le Marchand et al*.*^30^	✭	✭					✭		3
Chen et al*.*^23^	✭	✭		✭			✭		4
Landi et al*.*^13^	✭	✭	✭	✭	✭✭		✭		7
Chen et al*.*^22^	✭	✭	✭	✭			✭		5
Gao et al*.*^24^	✭	✭	✭	✭	✭✭		✭		7
Kiss et al*.*^11^	✭	✭		✭	✭✭		✭	✭	7
Kury et al*.*^15^	✭	✭	✭	✭	✭		✭		6
Cotterchio et al*.*^16^	✭	✭	✭		✭✭	✭	✭		7
Morita et al*.*^26^	✭	✭		✭	✭✭		✭	✭	7
Cleary et al*.*^28^	✭	✭	✭			✭	✭		5
Darazy et al*.*^29^	✭	✭	✭	✭	✭		✭	✭	7
Sameer et al*.*^27^	✭	✭		✭	✭✭	✭	✭	✭	8
Silva et al*.*^14^	✭	✭		✭			✭	✭	5
Saeed et al.^19^	✭	✭		✭	✭✭		✭		6
Chong et al*.*^17^	✭	✭		✭			✭	✭	5
Proenca et al.^8^	✭	✭	✭	✭	✭		✭	✭	7
Fernandes et al.^18^	✭	✭		✭			✭	✭	5
Kim et al.^25^	✭	✭		✭	✭✭		✭	✭	7

### Meta-analysis results: rs2031920

**Figure 2:**
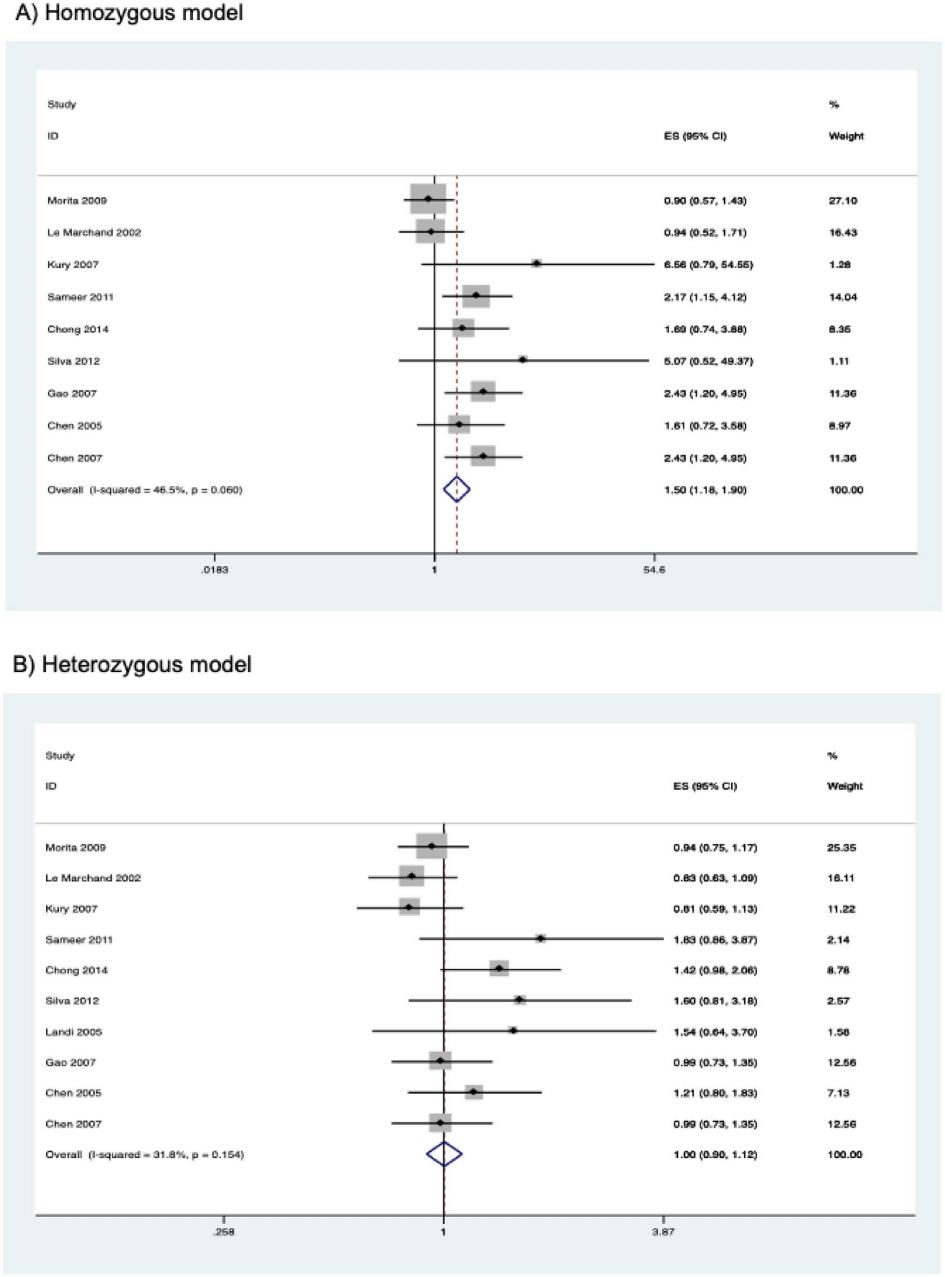

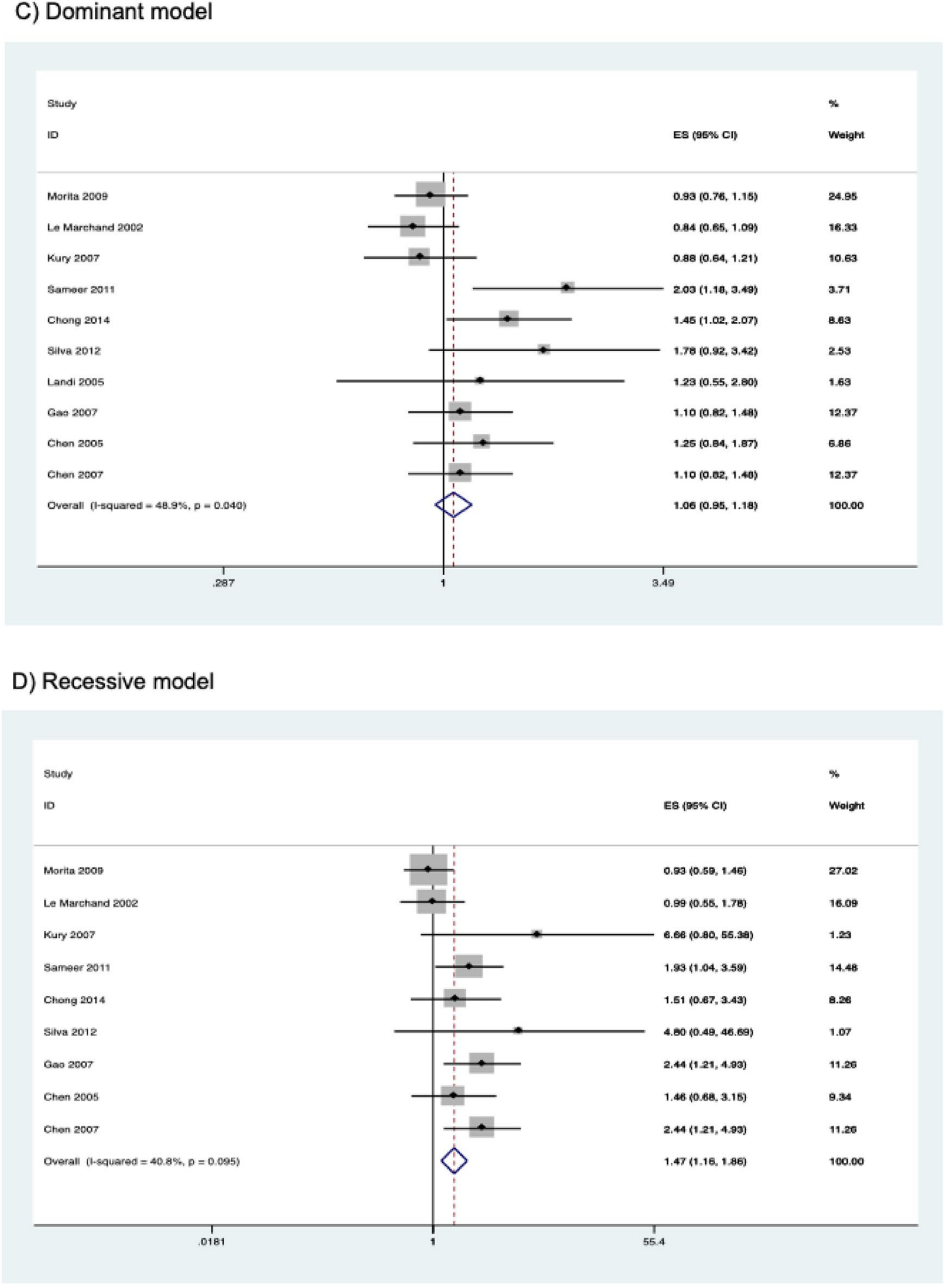

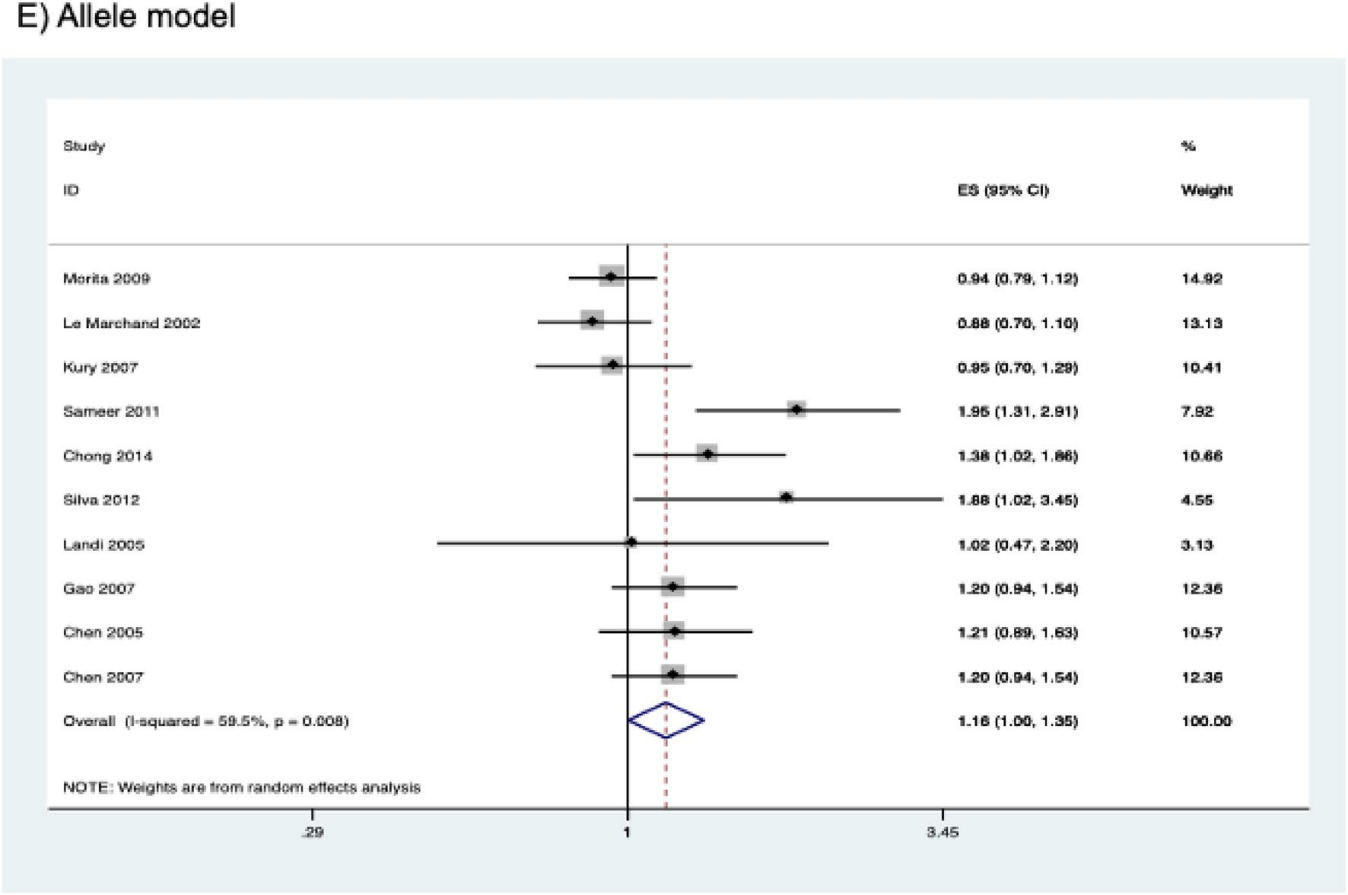
Forest plots of the association between *CYP2E1* rs2031920 polymorphism and the risk of colorectal cancer.

The results of the meta-analysis on the association of *CYP2E1* rs2031920 polymorphism with CRC risk are shown in Table 3. Overall, pooled results from 10 studies (comprising 3,695 cases and 4,887 controls) revealed a significant association between the polymorphism and an increased CRC risk in the homozygous (OR = 1.496, 95% CI   1.177–1.901, *P* = 0.001), recessive (OR = 1.467, 95% CI 1.160–1.857, *P* = 0.001), and allele models (OR = 1.162, 95% CI 1.001–1.349, *P* = 0.048) (Fig. 2). Subgroup analysis by ethnicity revealed that there were significant associations between *CYP2E1* rs2031920 polymorphism and CRC risk in Asians in homozygous (OR = 1.578, 95% CI 1.209–2.058, *P* = 0.001), recessive (OR = 1.526, 95% CI 1.176–1.980, *P* = 0.001), and allele models (OR = 1.231, 95% CI 1.031–1.469, *P* = 0.021), an observation consistent with the overall analysis (Table 3). In contrast, among Caucasians, only the homozygous and recessive models showed significant associations. Interestingly, the ORs of the associations were large in Caucasians (homozygous model, OR = 5.819, 95% CI 1.234–27.436; recessive model, OR = 5.720, 95% CI 1.214–26.954). No subgroup analysis was performed for other ethnicities as only one study was available for this subgroup^30^.

**Table 3:** Association between *CYP2E1* rs2031920 polymorphism and colorectal cancer risk.

Contrast model	Studies (N)	Cases (N)	Controls (N)	Model	OR (95% CI)	*P*
**Homozygous model**						
Overall	9	2582	3447	Fixed	1.496 (1.177–1.901)	0.001
Asian	6	1118	1757	Fixed	1.578 (1.209–2.058)	0.001
Caucasian	2	1059	1215	Fixed	5.819 (1.234–27.436)	0.026
High quality	7	2107	2789	Random	1.899 (1.220–2.954)	0.004
Low quality	2	475	658	Fixed	1.140 (0.709–1.834)	0.589
**Heterozygous model**						
Overall	10	3540	4722	Fixed	1.004 (0.899–1.121)	0.946
Asian	6	1585	2528	Fixed	1.058 (0.926–1.208)	0.406
Caucasian	3	1455	1581	Random	1.138 (0.686–1.887)	0.617
High quality	8	2913	3789	Fixed	1.027 (0.906–1.165)	0.673
Low quality	2	627	933	Random	0.969 (0.670–1.402)	0.868
**Dominant model**						
Overall	10	3695	4887	Fixed	1.059 (0.954–1.175)	0.285
Asian	6	1710	2663	Fixed	1.124 (0.992–1.275)	0.068
Caucasian	3	1464	1585	Fixed	1.027 (0.783–1.348)	0.846
High quality	8	3036	3909	Fixed	1.094 (0.971–1.233)	0.138
Low quality	2	659	978	Random	0.998 (0.679–1.467)	0.992
**Recessive model**						
Overall	9	3375	4626	Fixed	1.467 (1.160–1.857)	0.001
Asian	6	1710	2663	Fixed	1.526 (1.176–1.980)	0.001
Caucasian	2	1144	1324	Fixed	5.720 (1.214–26.954)	0.027
High quality	7	2716	3648	Fixed	1.599 (1.217–2.100)	0.001
Low quality	2	659	978	Fixed	1.142 (0.716–1.821)	0.578
**Allele model**						
Overall	10	7390	9774	Random	1.162 (1.001–1.349)	0.048
Asian	6	3420	5326	Random	1.231 (1.031–1.469)	0.021
Caucasian	3	2928	3170	Fixed	1.079 (0.834–1.397)	0.562
High quality	8	6072	7818	Random	1.216 (1.020–1.450)	0.030
Low quality	2	1318	1956	Random	1.013 (0.743–1.380)	0.936

Similarly, subgroup analysis by study quality also revealed statistically significant results in the homozygous (OR = 1.899, 95% CI 1.220–2.954, *P* = 0.004), recessive (OR = 1.599, 95% CI 1.217–2.100, *P* = 0.001) and allele models (OR = 1.216, 95% CI 1.020–1.450, *P* = 0.030) in the high quality studies. On the other hand, no significant association between rs2031920 and the risk of CRC was observed in the low quality studies under all five genetic models (*P* > 0.05; Table 3).

### Meta-analysis results: rs3813867

**Figure 3:**
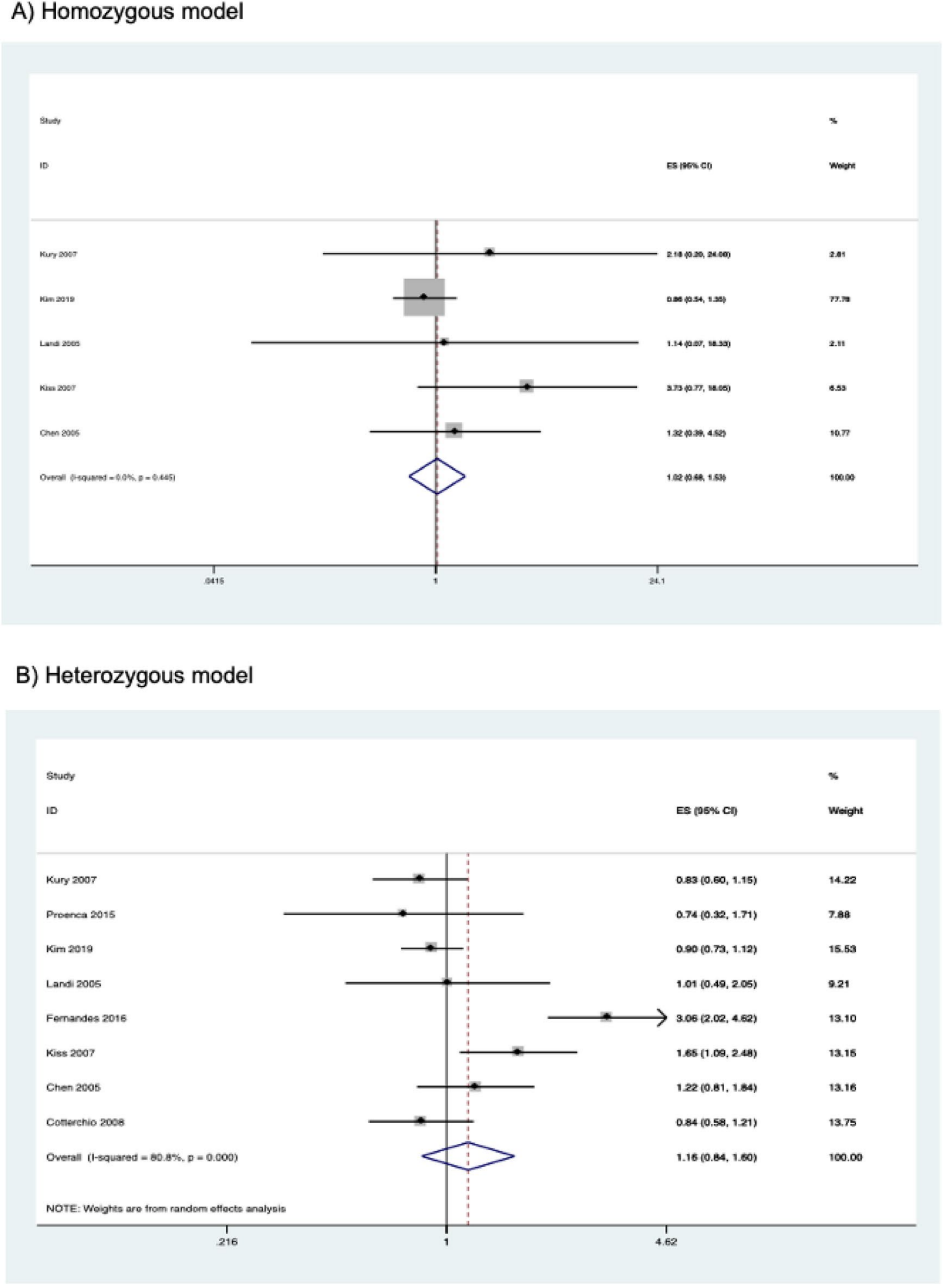

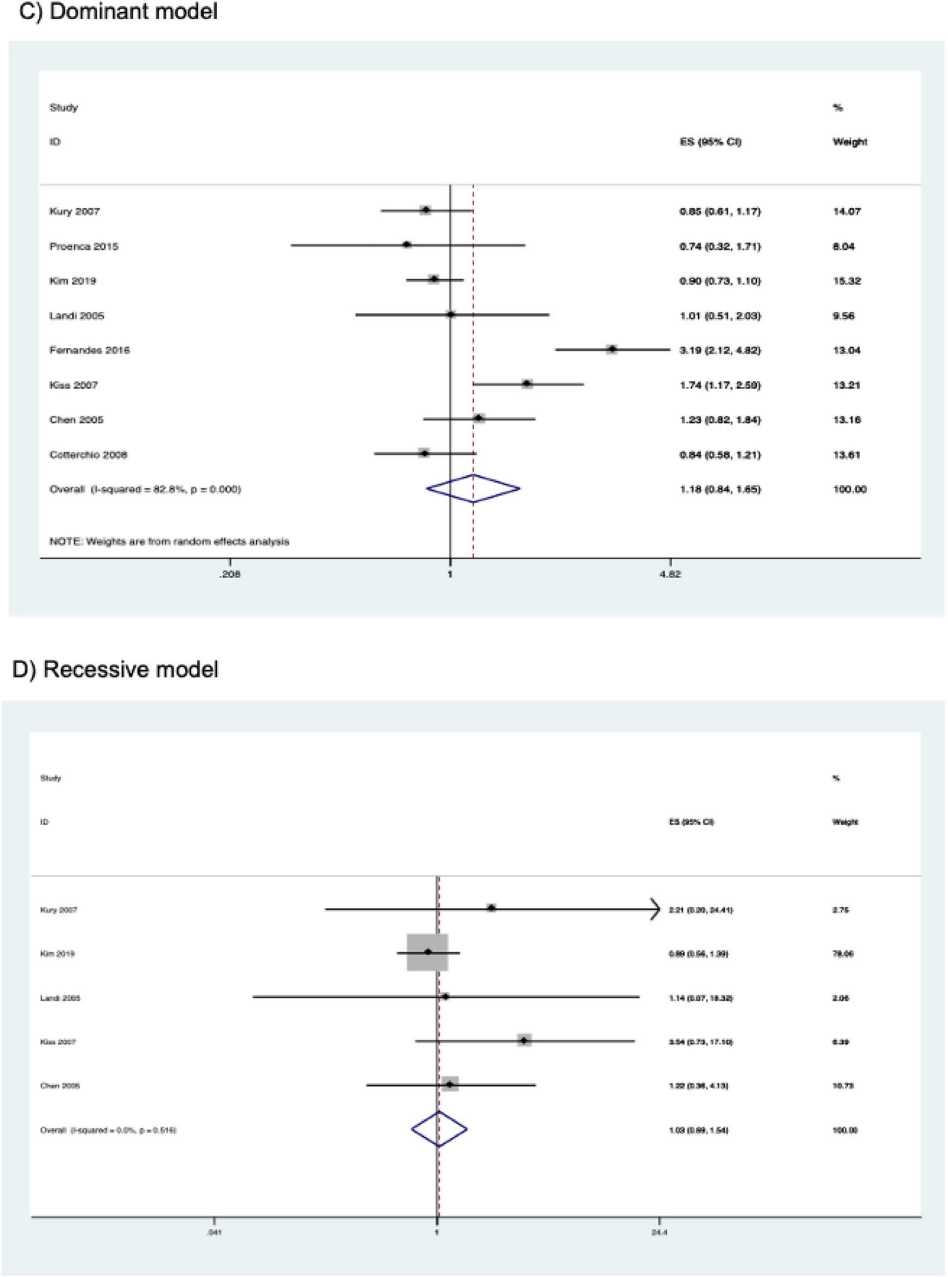

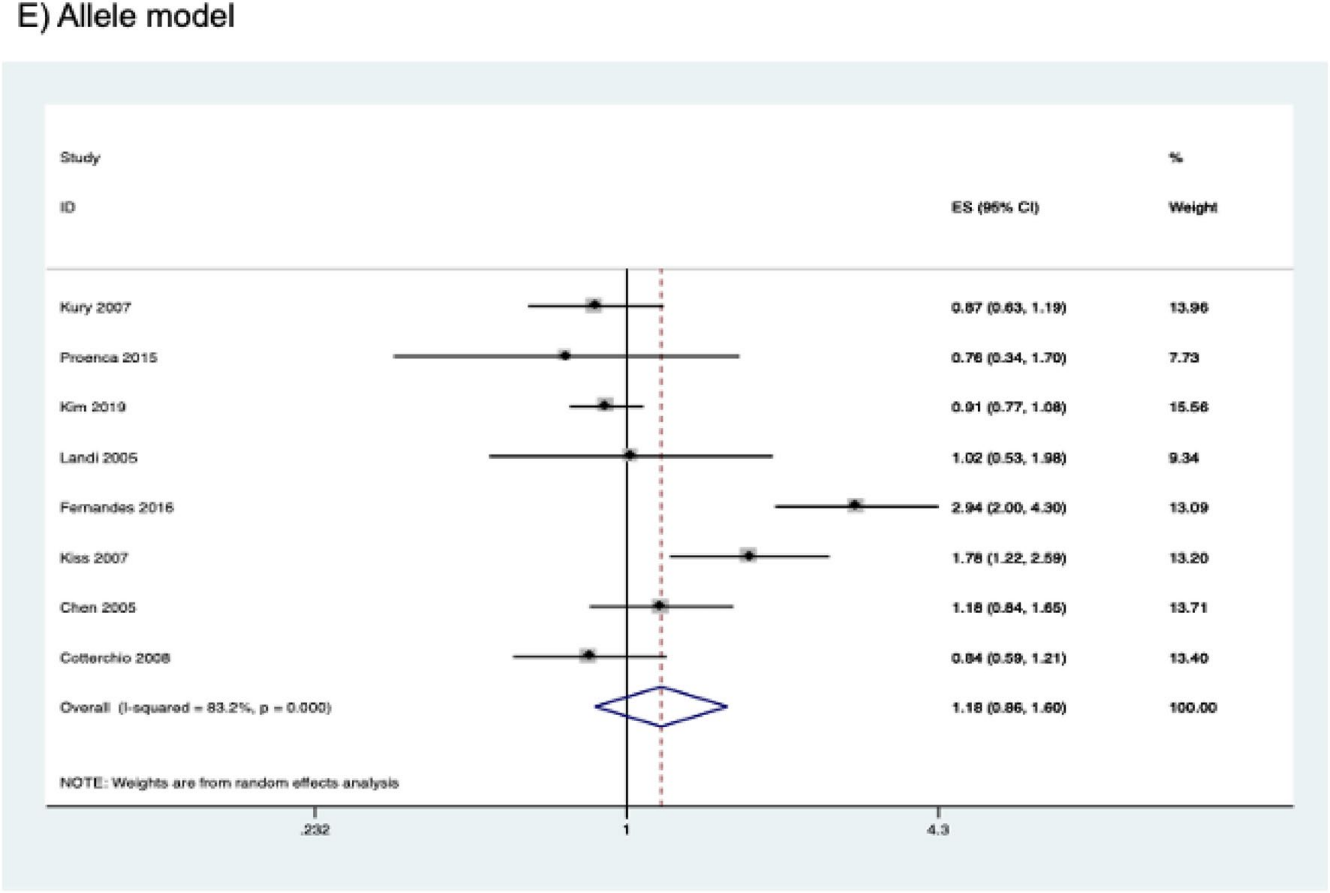
Forest plots of the association between *CYP2E1* rs3813867 and colorectal cancer risk.

Pooled results from eight studies (comprising 4,055 cases and 4,801 controls) are shown in Table 4. The *CYP2E1* rs3813867 polymorphism was not significantly associated with CRC risk in any of the genetic models studied (homozygous model, OR = 1.020, 95% CI 0.682–1.526, *P* = 0.923; heterozygous model, OR = 1.161, 95% CI 0.841–1.603, *P* = 0.366; dominant model, OR = 1.179, 95% CI 0.845–1.645, *P* = 0.333; recessive model, OR = 1.302, 95% CI 0.693–1.538, *P* = 0.876; allele model, OR = 1.175, 95% CI 0.862–1.602, *P* = 0.306) (Fig. 3). Subgroup analysis by ethnicity and study quality also revealed no significant association (Table 4).

**Table 4:** Association between *CYP2E1* rs3813867 polymorphism and colorectal cancer risk.

Contrast model	Studies (N)	Cases (N)	Controls (N)	Model	OR (95% CI)	*P*
**Homozygous model**						
Overall	5	2425	2475	Fixed	1.020 (0.682–1.526)	0.923
Asian	2	760	663	Fixed	0.903 (0.588–1.385)	0.639
Caucasian	3	1665	1812	Fixed	2.628 (0.799–8.647)	0.112
High quality	4	2342	2258	Fixed	0.989 (0.645–1.514)	0.958
**Heterozygous model**						
Overall	8	3992	4754	Random	1.161 (0.841–1.603)	0.366
Asian	2	1060	953	Fixed	0.965 (0.797–1.168)	0.716
Caucasian	4	2634	3202	Random	1.026 (0.728–1.446)	0.885
Other ethnicity	2	298	599	Random	1.579 (0.395–6.317)	0.519
High quality	7	3857	4424	Random	1.150 (0.794–1.667)	0.459
**Dominant model**						
Overall	8	4055	4801	Random	1.179 (0.845–1.645)	0.333
Asian	2	1110	996	Fixed	0.958 (0.798–1.150)	0.643
Caucasian	4	2644	3,206	Random	1.052 (0.727–1.522)	0.789
Other ethnicity	2	301	599	Random	1.612 (0.386–6.733)	0.513
High quality	7	3,916	4463	Random	1.170 (0.798–1.717)	0.421
**Recessive mode**l						
Overall	5	2922	2955	Fixed	1.032 (0.693–1.538)	0.876
Asian	2	1110	996	Fixed	0.920 (0.603–1.405)	0.701
Caucasian	3	1812	1959	Fixed	2.558 (0.778–8.414)	0.122
High quality	4	2783	2617	Fixed	1.011 (0.663–1.542)	0.958
**Allele mode**l						
Overall	8	8110	9602	Random	1.175 (0.862–1.602)	0.306
Asian	2	2220	1992	Fixed	0.960 (0.823–1.119)	0.599
Caucasian	4	5288	6412	Random	1.071 (0.735–1.562)	0.720
Other ethnicity	2	602	1198	Random	1.564 (0.415–5.897)	0.509
High quality	7	7832	8926	Random	1.174 (0.817–1.687)	0.387

### Meta-analysis results: rs6413432

**Figure 4:**
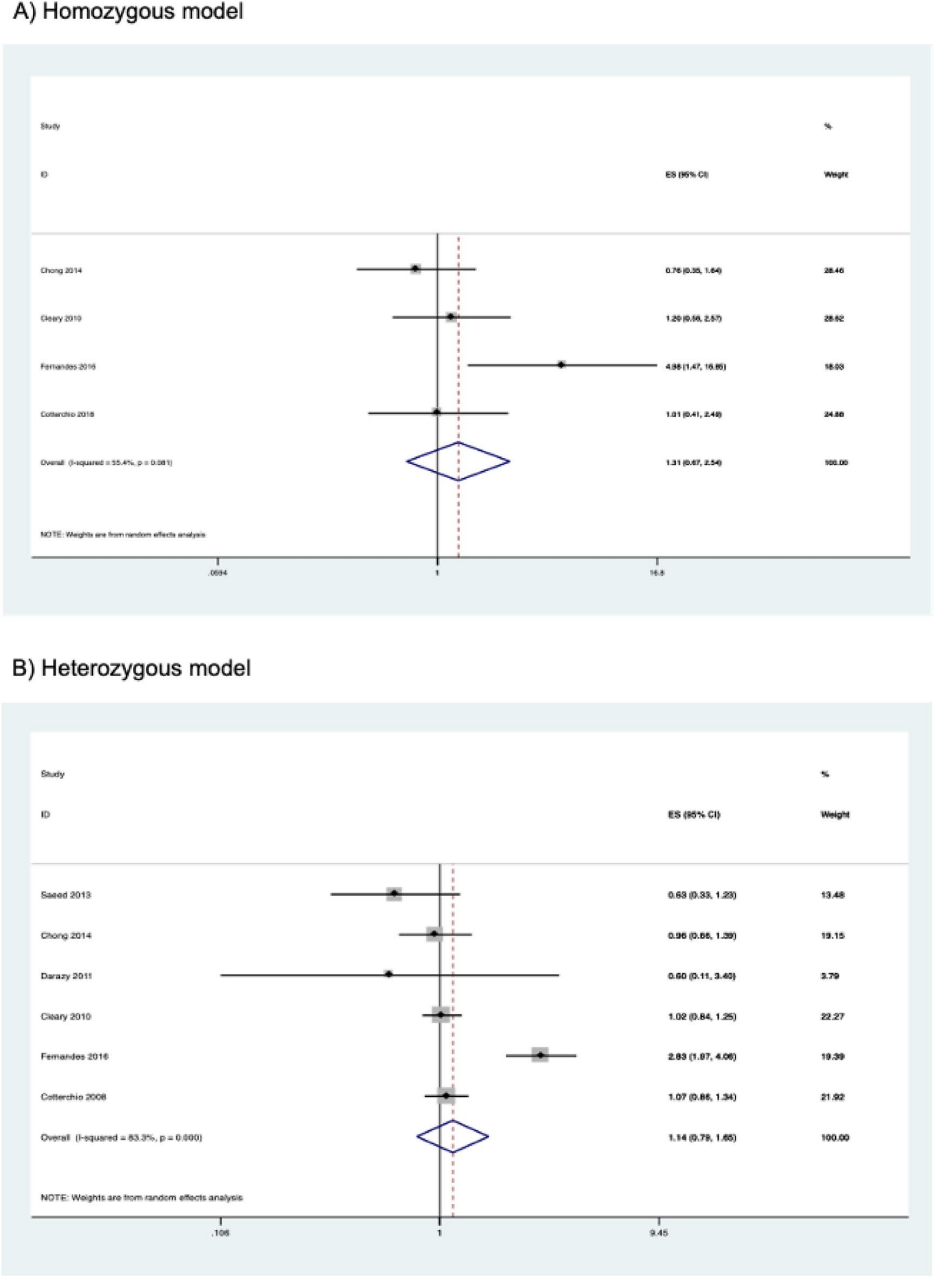

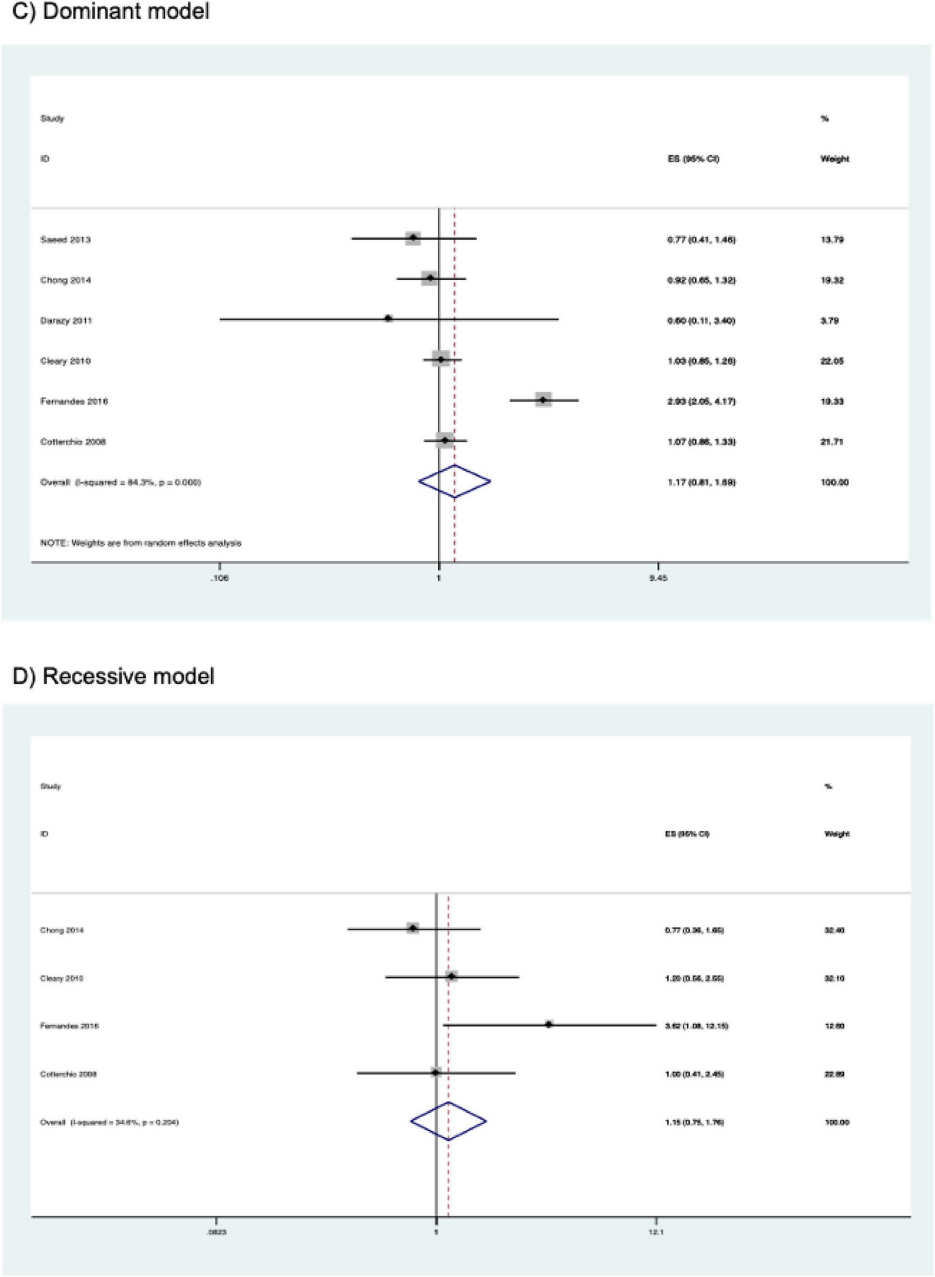

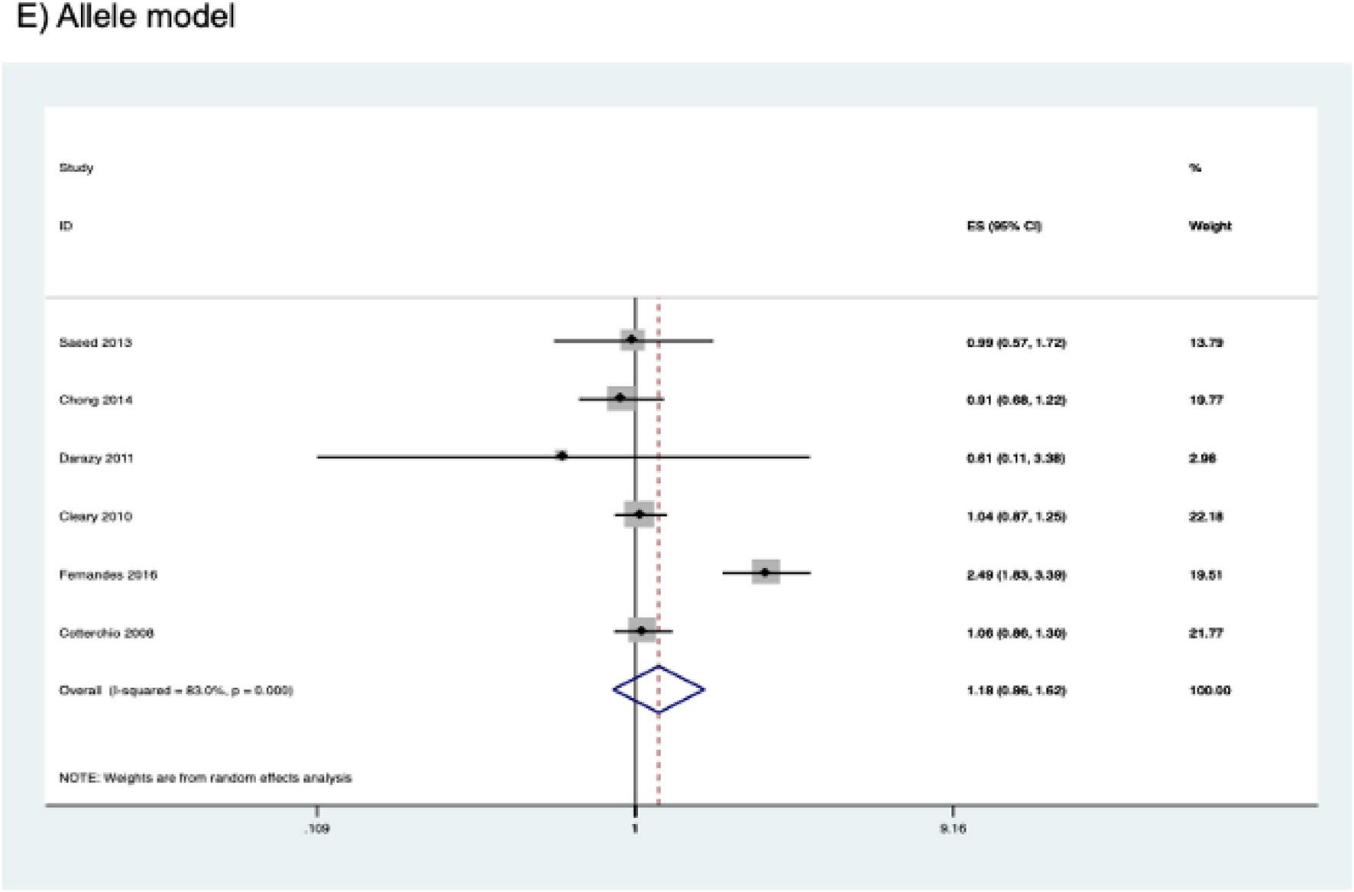
Forest plots of the association between *CYP2E1* rs6413432 polymorphism and colorectal cancer risk.

The association of the *CYP2E1* rs6413432 polymorphism with susceptibility to CRC is shown in Table 5. The combined results from six case–control studies involving 2,552 cases and 3,608 controls showed that there was no significant association between the polymorphism and susceptibility to CRC in all genetic models studied. The combined ORs and their 95% CIs were as follows: homozygous model (OR = 1.307, 95% CI 0.673–2.540, *P* = 0.429); heterozygous model (OR = 1.142, 95% CI 0.790–1.650, *P* = 0.481); dominant model (OR = 1.172, 95% CI 0.811–1.694, *P* = 0.399); recessive model (OR = 1.146, 95% CI 0.745–1.762, *P* = 0.535); allele model (OR = 1.177, 95% CI 0.858–1.616, *P* = 0.313) (Table 5 and Fig. 4). No significant association was observed even after performing subgroup analysis by ethnicity. Subgroup analysis by study quality was not performed because all studies were of high quality.

**Table 5:** Association between *CYP2E1* rs6413432 polymorphism and colorectal cancer risk.

Contrast model	Studies (N)	Cases (N)	Controls (N)	Model	OR (95% CI)	*P*
**Homozygous model**						
Overall	4	1866	2737	Random	1.307 (0.673–2.540)	0.429
Caucasian	2	1612	2065	Fixed	1.118 (0.626–1.998)	0.707
**Heterozygous model**						
Overall	6	2508	3545	Random	1.142 (0.790–1.650)	0.481
Caucasian	3	2034	2584	Fixed	1.041 (0.896–1.208)	0.600
Other ethnicity	2	308	475	Random	1.376 (0.319–5.940)	0.669
**Dominant model**						
Overall	6	2552	3608	Random	1.172 (0.811–1.694)	0.399
Caucasian	3	2056	2609	Fixed	1.044 (0.903–1.209)	0.559
Other ethnicity	2	321	479	Random	1.546 (0.420–5.695)	0.513
**Recessive model**						
Overall	4	2401	3459	Fixed	1.146 (0.745–1.762)	0.535
Caucasian	2	1999	2539	Fixed	1.109 (0.621–1.980)	0.727
**Allele model**						
Overall	6	5104	7216	Random	1.177 (0.858–1.616)	0.313
Caucasian	3	4112	5218	Fixed	1.044 (0.912–1.194)	0.535
Other ethnicity	2	642	958	Random	1.617 (0.655–3.996)	0.297

### Sensitivity analysis

Sensitivity analysis was performed by sequentially omitting individual studies to assess the stability of the results. For rs2031920, the results of the homozygous, recessive, and dominant models were not altered with the omission of any individual study (Supplementary Information online). However, for the heterozygous model, omitting^13,14,17,22,27^ changed the results from non-significant to significant. However, this change was not unexpected, as the combined results of rs2031920 under the heterozygous model were at the borderline OR value (OR = 1.004; Table 3). A similar observation was noted in the allele model, where removal of several studies changed the results from non-significant to significant. Similar to the heterozygous model, the instability of the results for the allele model was not unexpected, as the lower limit of the 95% CI was 1.001, which is also a borderline value.

For rs3813867, the result for the homozygous model was also unstable for the same reason. However, for the heterozygous and recessive models, the results appeared to be largely driven by Fernandes et al*.*^18^and Kiss et al.^11^, respectively (Supplementary Information online). Indeed, the omission of Fernandes et al*.*^18^ also significantly altered the results of the homozygous and recessive models of rs6413432 (Supplementary Information online). For all other genetic models, the results did not change when any of the studies was omitted.

### Publication bias diagnosis

**Figure 5:**
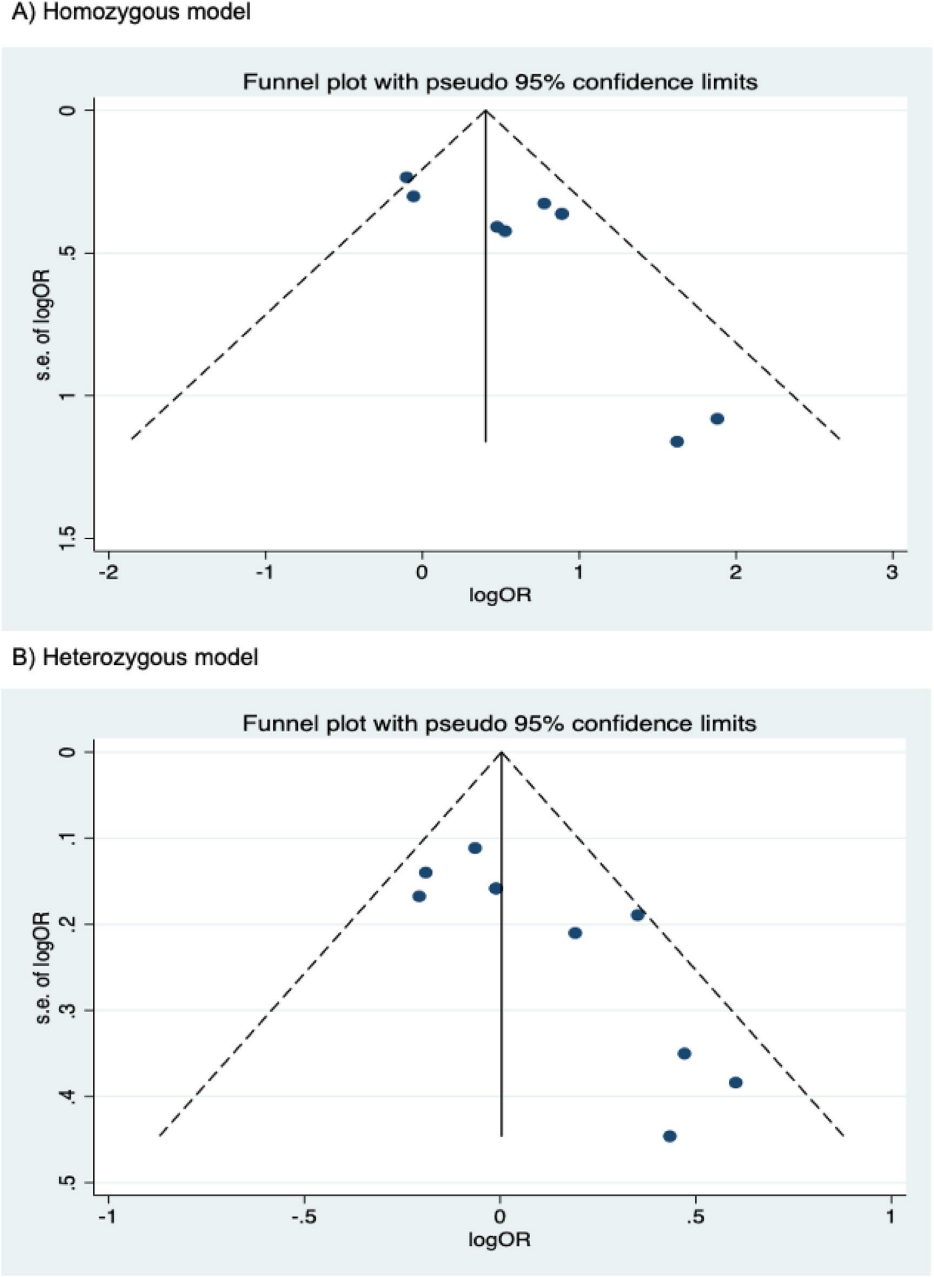

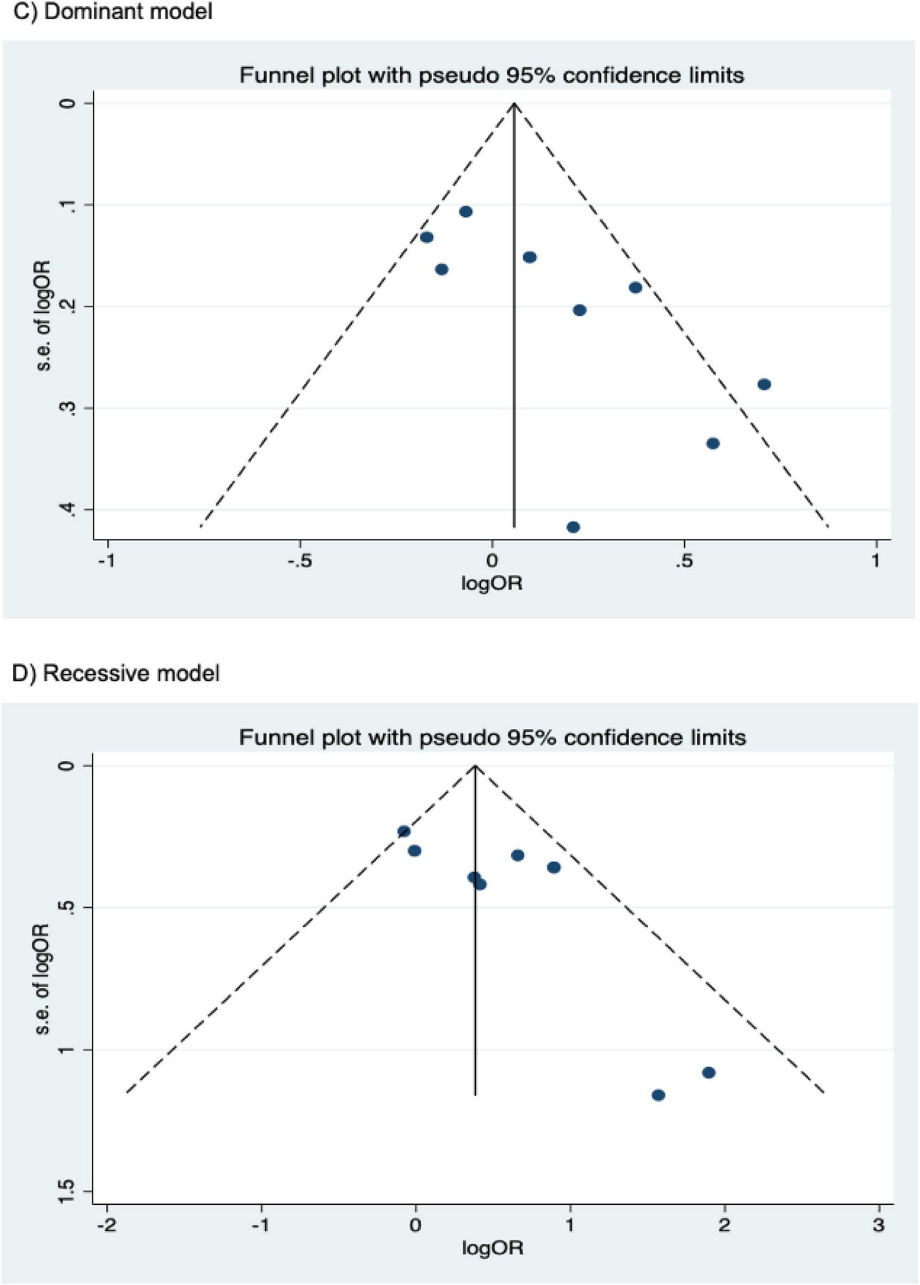

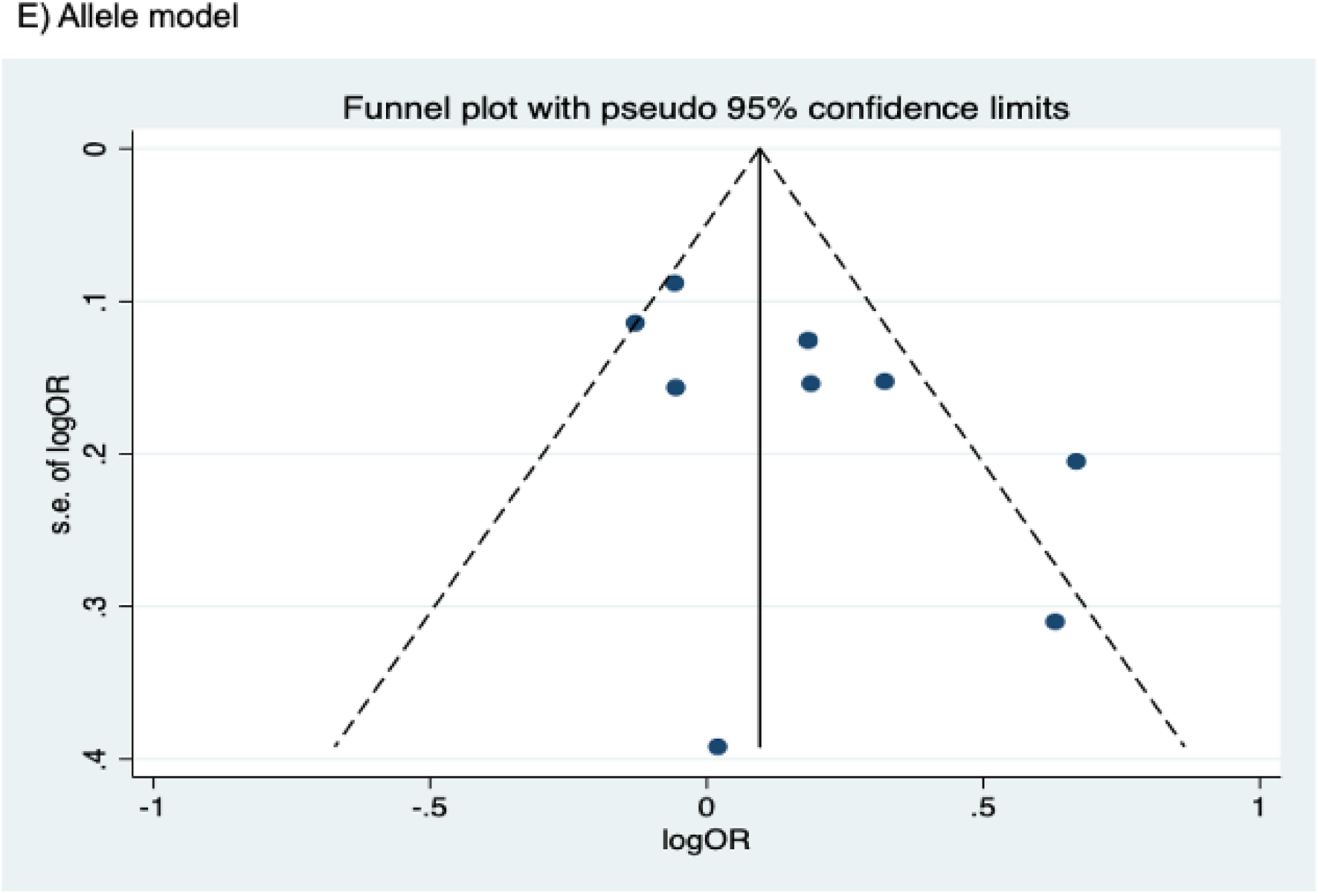
Funnel plots of *CYP2E1* rs2031920 polymorphism and colorectal cancer risk.

**Figure 6:**
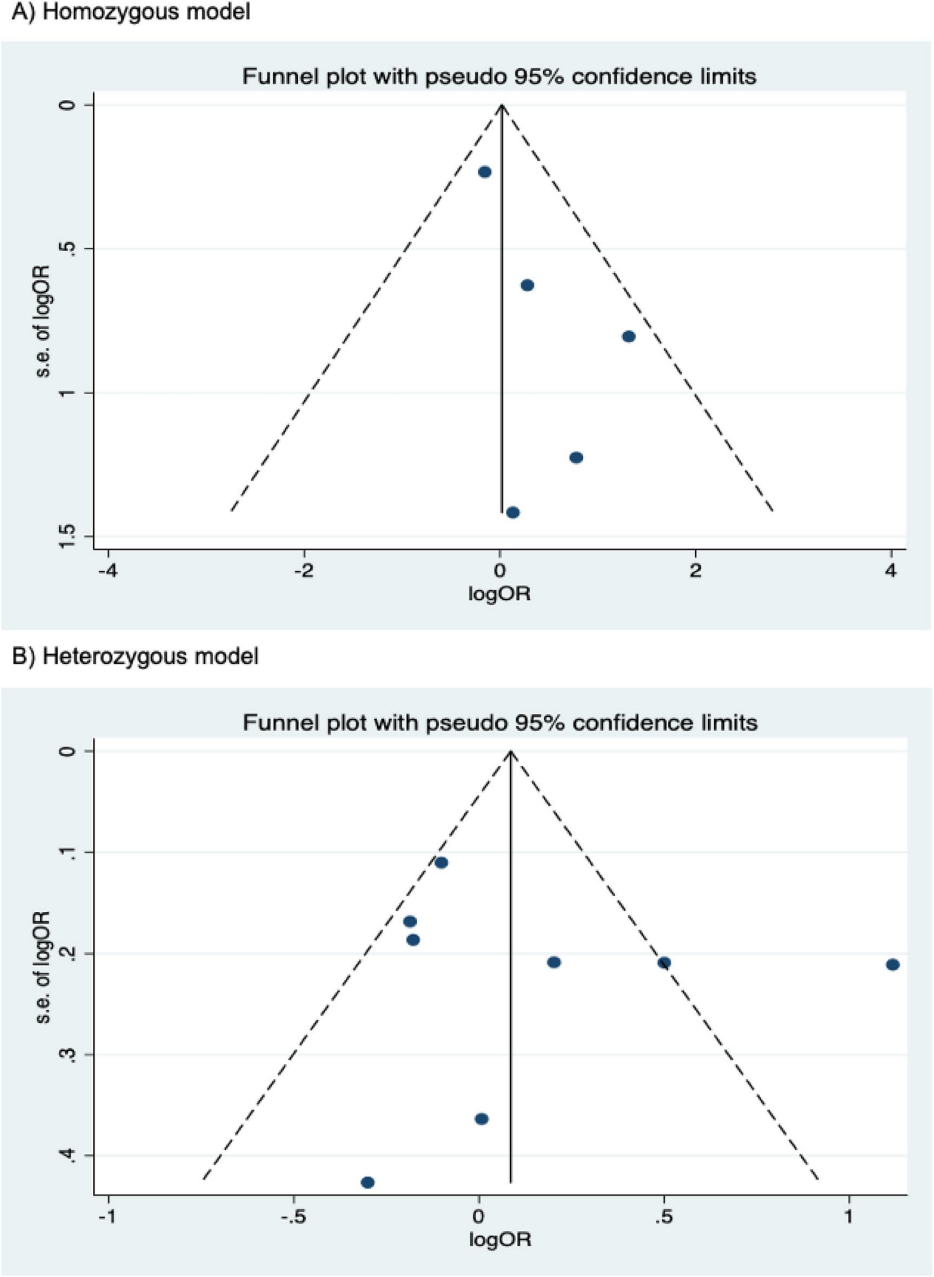

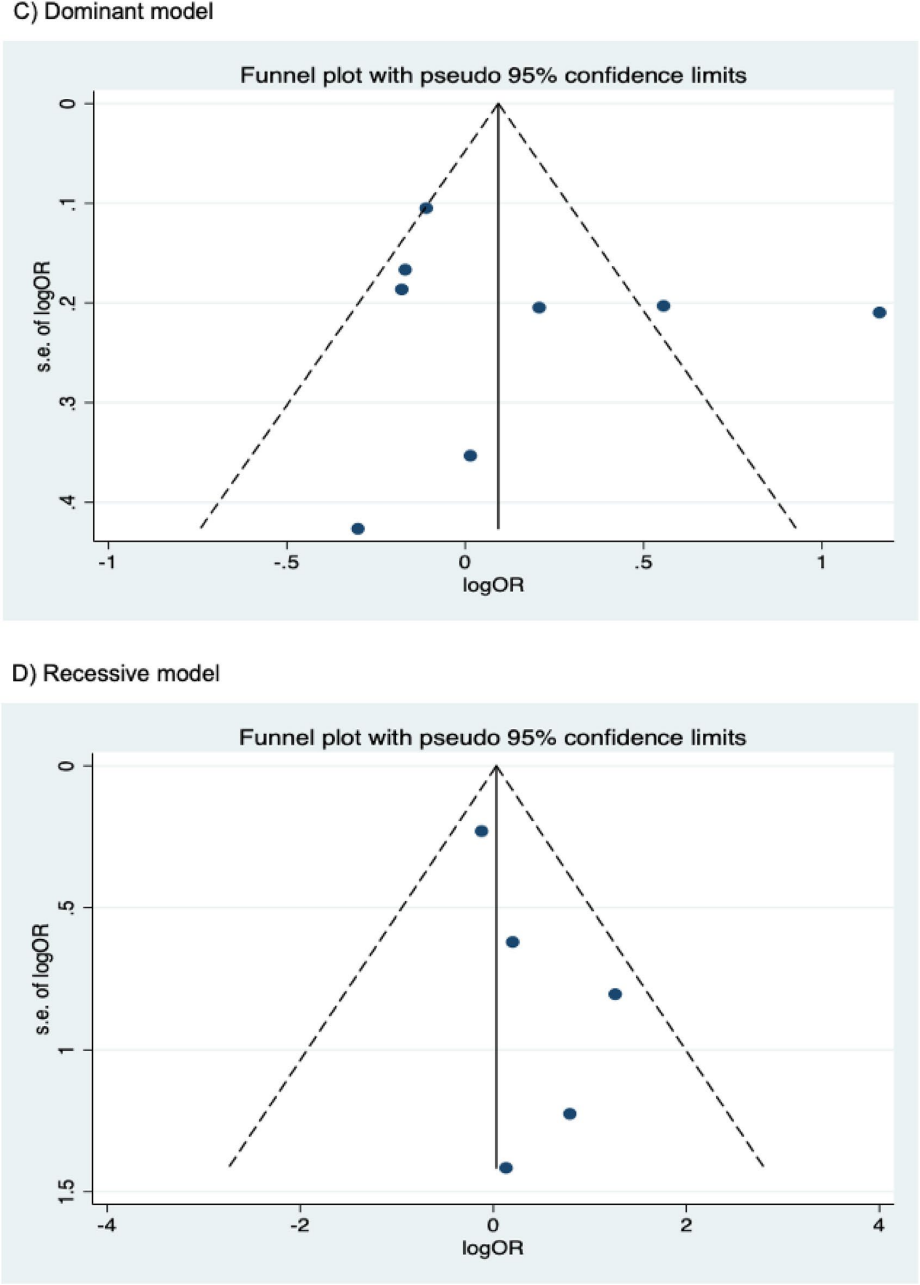

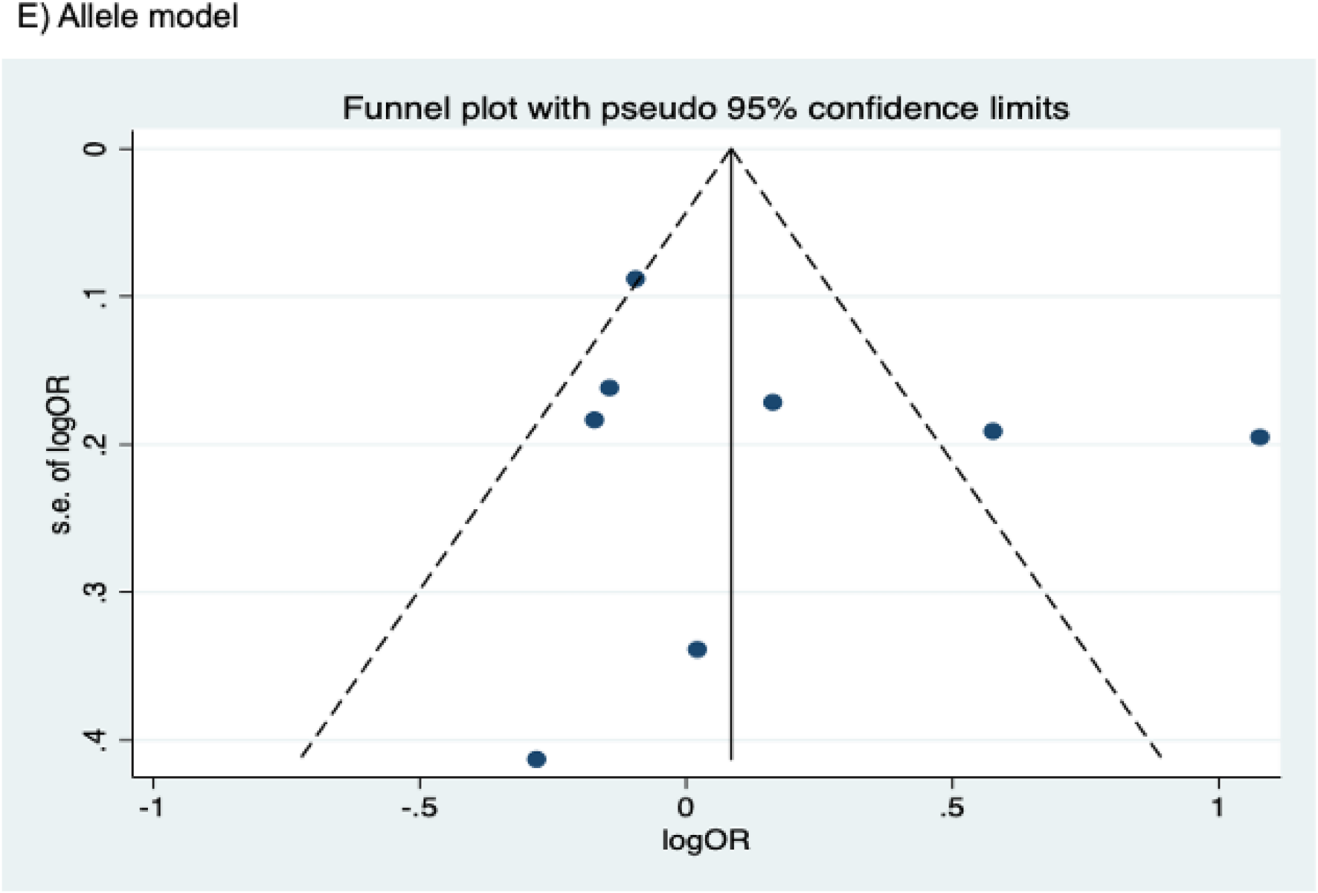
Funnel plots of *CYP2E1* rs3813867 polymorphism and colorectal cancer risk.

**Figure 7:**
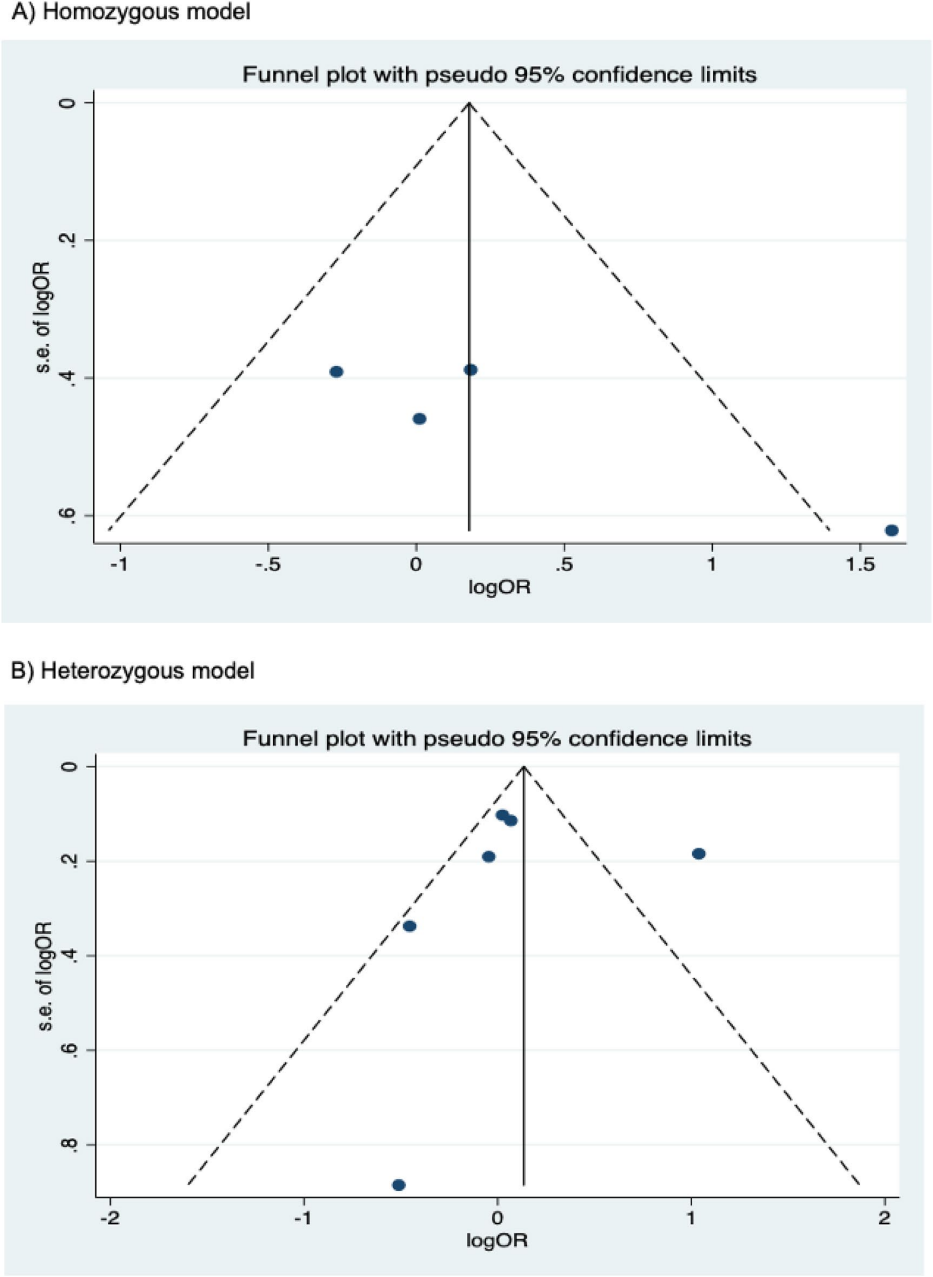

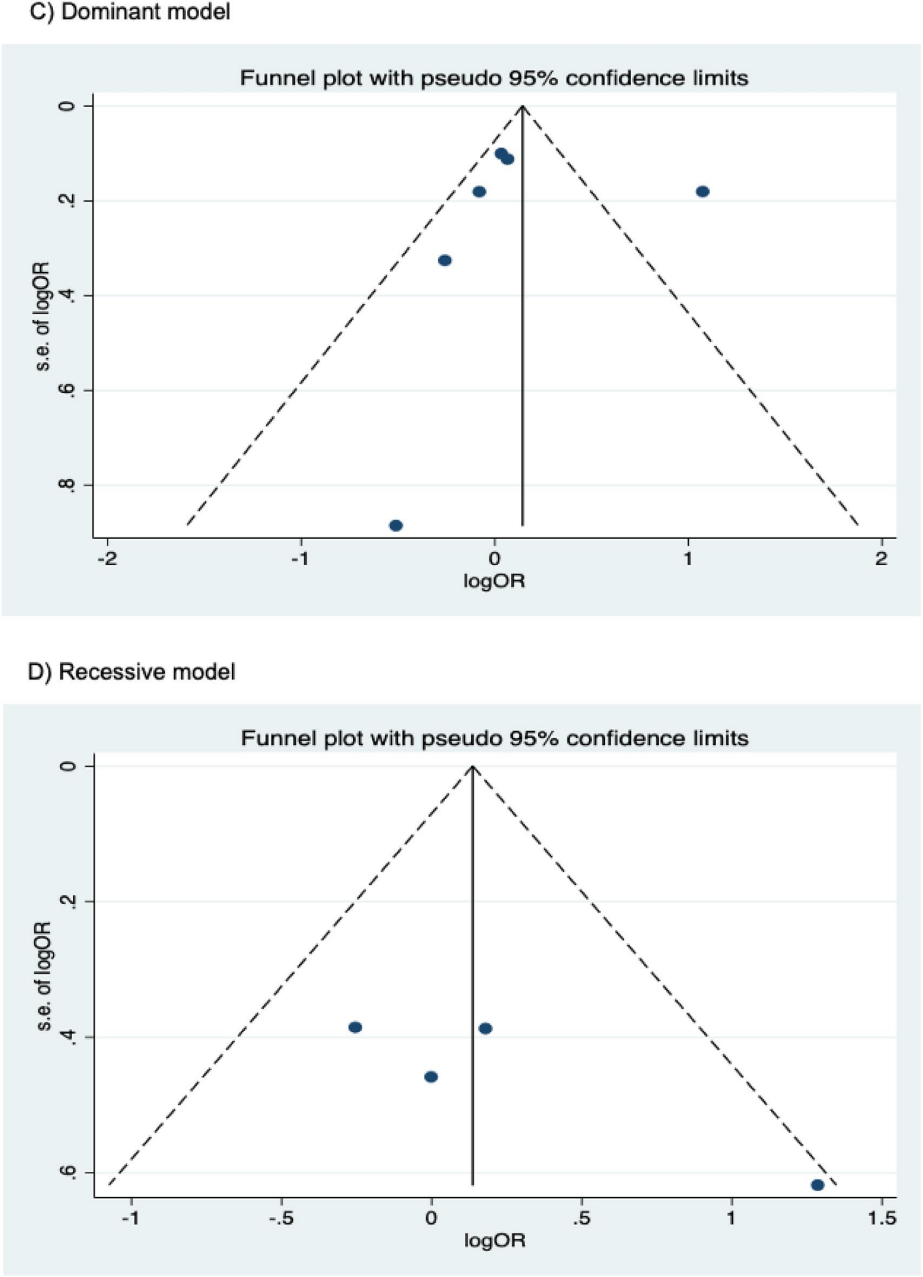

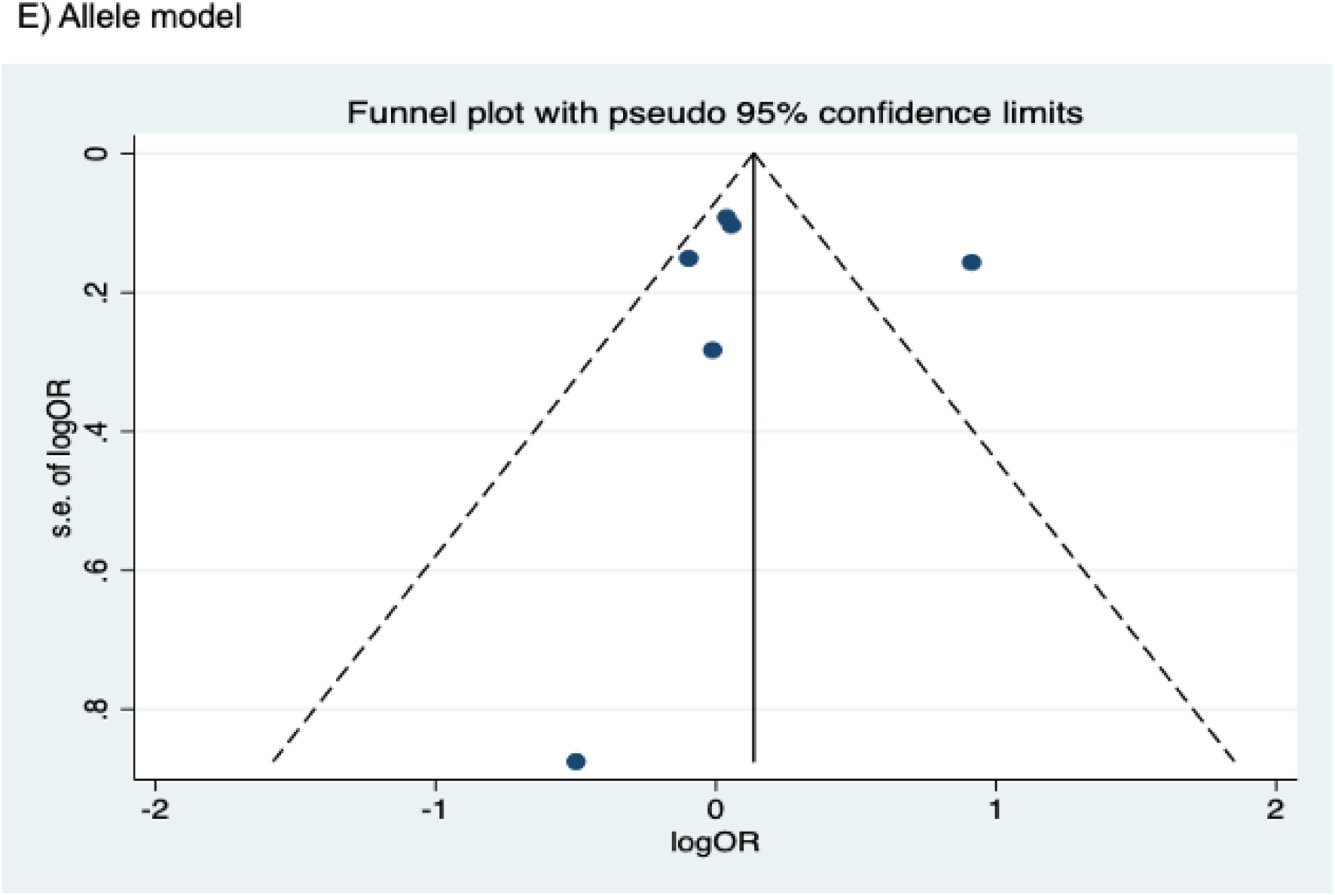
Funnel plots of *CYP2E1* rs6413432 polymorphism and colorectal cancer risk.

The presence of publication bias was examined using Begg’s and Egger’s tests and visually verified using funnel plots (Figs. 5, 6, 7). For rs2031920, no significant publication bias was detected in the allele model (*P* > 0.05 for both Begg’s and Egger’s tests). However, both tests revealed a significant publication bias in the heterozygous (Begg's test *P* = 0.032, Egger's test *P* = 0.012) and dominant models (Begg's test *P* = 0.032, Egger's test *P* = 0.018). Apart from this, no publication bias was observed in the homozygous and recessive models in Begg’s test but appeared to be significant in Egger’s test (homozygous, Begg’s test *P* = 0.251, Egger’s test *P* = 0.035; recessive, Begg’s test *P* = 0.175, Egger’s test *P* = 0.037). ‘Trim and fill’ analysis was performed for all four genetic models that showed significant publication bias in at least one of the tests. The homozygous, heterozygous, dominant, and recessive models were found to have four, three, five, and four missing studies, respectively. However, imputation of these missing studies did not significantly change the results (homozygous, *P* = 0.052; heterozygous, *P* = 0.583; dominant, *P* = 0.341; recessive, *P* = 0.079).

For the rs3813867 polymorphism, no significant publication bias was detected by both the Begg’s and Egger’s tests (homozygous, Begg’s test *P* = 0.624, Egger’s test *P* = 0.141; heterozygous, Begg’s test *P* = 0.083, Egger’s test *P* = 0.541; dominant, Begg’s test *P* = 0.138, Egger’s test *P* = 0.306). Similarly, no publication bias was detected for rs6413432 (homozygous, Begg’s test *P* = 0.497, Egger’s test *P* = 0.113; heterozygous, Begg’s test *P* = 0.851, Egger’s test *P* = 0.957; dominant, Begg’s test *P* = 0.348, Egger’s test *P* = 0.963; recessive, Begg’s test *P* = 0.174, Egger’ test *P* = 0.128; allele, Begg’s test *P* = 0.348, Egger’s test *P* = 0.912).

## Discussion

*CYP2E1*, located on chromosome 10q26.3, encodes the CYP2E1 enzyme that is mainly localized in the liver. CYP2E1 belongs to the phase I group of drug-metabolizing enzymes that are involved in the metabolism of several small molecules such as ethanol, acetaminophen and procarcinogens like nitrosamines and azo compounds^31^. The enzyme has been extensively studied as it is directly involved in the metabolic activation of more than 85 xenobiotics to hepatotoxic or carcinogenic metabolites^32^. In addition, CYP2E1 is known to be the most active CYP450 isoenzyme because of its ability to reduce molecular oxygen to highly reactive oxygen species (ROS) even in the absence of a substrate^33^. Excessive levels of the ROS accelerate cancer development by acting on messengers in intracellular signaling pathways, leading to activation of lipid peroxidation, DNA damage, and carcinogenesis^34^. For these reasons, *CYP2E1* is one of the most intensively studied cytochrome genes in cancer^35^.

Over the decades, several studies have focused on a few important polymorphisms of *CYP2E1* that can potentially affect the function of the gene. These include the rs2031920 and rs3813867 polymorphisms, which are located in the 5'-regulatory region of *CYP2E1*, as well as the rs6413432 polymorphism, which is located in intron 6 of the gene^36^. The rs2031920 polymorphism has been associated with higher transcriptional and enzymatic activity due to the replacement of cytosine with thymine at position 1,019 of the gene^37^. Meanwhile, the rs3813867 polymorphism of *CYP2E1* results from the substitution of guanine with cytosine at the 1259th position, whereas rs6413432 involves a substitution of thymine with adenine at the 7678th position of the gene^38^. These substitutions may lead to altered binding affinity of transcription factors and other regulatory elements, causing changes in the amount of protein product and subsequently the risk of cancer^39^.

Despite being extensively investigated, the association between *CYP2E1* polymorphisms and CRC risk remains inconclusive, as conflicting results have been reported in different studies. These conflicting results can be attributed to numerous factors, including the sample size of individual studies, ethnicity of study participants, geographical variations, as well as environmental factors such as dietary habits^5^. To address these discrepancies, we conducted a systematic review and meta-analysis to combine the results of previous studies, in order to yield a more accurate estimation on the association between *CYP2E1* polymorphisms and CRC risk. In contrast to a pooled analysis, a meta-analysis considers the characteristics of individual studies and weighs them appropriately based on well-accepted statistical parameters, such as sample size, before combining them, thereby reducing the potential for erroneous conclusions^40^. We demonstrated a statistically significant association of the *CYP2E1* rs2031920 polymorphism with CRC risk under the homozygous (OR = 1.496, 95% CI 1.177–1.901, *P* = 0.001), recessive (OR = 1.467, 95% CI 1.160–1.857, *P* = 0.001) and allele (OR = 1.162, 95% CI 1.001–1.349, *P* = 0.048) models. This observation may be attributed to the location of the polymorphism, which falls within the transcriptional regulatory region of *CYP2E1*. Therefore, the nucleotide substitution of this polymorphism could affect the binding of transcription factors to the 5'-flanking region of *CYP2E1*, thereby altering its mRNA expression levels^41^. The positive association between rs2031920 polymorphism and cancer susceptibility has also been studied in tumor types other than CRC, including cancers of the head and neck^42,43^, esophagus^41^, lung^44^, stomach^45^, urological organs^46^, and urinary tract^47^. However, there were also a few studies suggesting the opposite association, whereby the rs2031920 polymorphism may serve as a protective factor as in the case of nasopharyngeal cancer in the Tunisia populations^48^ and bladder cancer in Asians^49^. These observations suggest that the distribution of allele or genotype frequency varies in different populations and association may be different between cancer types. Therefore, we stratified our meta-analysis by ethnicity to gain better insight into the impact of ethnic diversity on the association of these polymorphisms with the risk of CRC. That being said, subgroup analyses revealed slight differences in risk association between Asians and Caucasians. This could be explained by the higher allele frequency of the rs2031920 c2 allele in the Asian population compared to the Caucasian population, which is consistent with the observation of Wang et al*.*^9^ that different ethnic groups generally have not only differences in the living environment, dietary habits, and genetic backgrounds, but also in the frequency distribution of *CYP2E1* genotypes.

The results of this meta-analysis suggested that there was no significant association of the rs3813867 polymorphism with susceptibility to CRC under all genetic models examined. Our finding was in agreement with previous studies that also found no association with CRC risk in study samples from Australia^12^, Spain^13^ and Brazil^8^. In contrast, a significant association was found in the study by Kiss et al*.*^11^, whereas other studies by Kury et al*.*^15^ and Kim et al*.*^25^ found a positive association between the polymorphism and CRC risk only in individuals who regularly consumed red meat. These discrepancies highlight the possible existence of gene–gene or gene-environment interactions in influencing the effects of genetic polymorphisms on CRC risk^50,51^.

Similarly, no significant association was observed between rs6413432 polymorphism and CRC risk in our study, which is consistent with studies in other populations, such as in Lebanese by Darazy et al*.*^29^, in Saudi Arabians by Saeed et al*.*^19^, and in Malaysians by Chong et al*.*^17^. In other cancers, several previous studies also supported our findings, showing a non-significant association between the rs6413432 polymorphism and susceptibility to urinary cancer^47^ and gastric cancer^52^. In contrast to the aforementioned rs2031920 polymorphism, the functional effect of rs6413432 has not been conclusively proven, but it is thought to enhance transcriptional activity and affect *CYP2E1* expression and the catalytic activity of the encoded enzyme^53^. Nevertheless, further studies are needed to increase statistical power to detect and confirm even the slightest effect of the rs6413432 polymorphism on the risk of CRC^54^.

The results of our meta-analysis were consistent with previous meta-analyses by Peng et al*.*^20^ and Jiang et al.^21^. However, because more studies were used (and more participants – a total of 23,598 subjects – were included) for analysis in the present work, our study power is higher and the risk estimate is therefore more reliable. In addition, the meta-analysis by Jiang et al*.*^21^ was limited to participants from Western populations only, and no study represented the Asian population. Thus, the results of our study are more representative of the global population. This, together with the inclusion of recent studies in this area of research, allows us to present the most up-to-date summary and assessment of the associations between the three *CYP2E1* polymorphisms and the risk of CRC. Apart from that, unlike the previous meta-analyses by Jiang et al*.*^21^ and Peng et al*.*^20^, the current meta-analysis used Scopus as one of the databases to search for relevant articles. In general, Scopus includes a wider range of journals and also contains more articles than the Web of Science^55^, allowing more relevant studies to be identified and included in the analysis. We also performed an additional stratified analysis based on study quality using the Newcastle–Ottawa scale to provide a comprehensive picture of the evidence based on all included studies. These are the strengths of the present work.

However, there are also several limitations that need to be acknowledged in the present study. For instance, the modest number of included studies might still be insufficient to find a significant association between the rs3813867 and rs6413432 polymorphisms and the risk of CRC, although the power to detect a significant association was improved by this meta-analysis. In addition, there is a lack of ethnic diversity as there were no data on African populations in these eighteen studies, which focused mainly on Asians and Caucasians. Another limitation is the concern for the occurrence of publication bias, as only published studies were included. Nevertheless, imputation using the ‘trim-and-fill’ analysis showed that the results are unlikely to change even in the absence of publication bias. Finally, gene–gene or gene-environment interactions, which are known to also contribute to the risk of CRC, were not examined in the present work because of the lack of information in the included studies. However, the lack of gene–gene or gene-environment studies does not change the fact that a single gene can influence the risk of CRC, albeit modestly, as has been demonstrated in many other studies^56–62^. The reproducibility of the study results (both with individual studies and with previous meta-analyses) suggests that the study result was not likely due to chance alone. Although positive results from a single gene cannot usually be translated into clinical practice, knowledge of which low penetrance polymorphisms might influence the risk of CRC may shed light on which genetic pathways to focus on in designing a genetic screening panel in the future, which can undoubtedly contribute to a more individualized approach to medicine^63^.

In conclusion, the results of this meta-analysis suggest that the *CYP2E1* rs2031920 polymorphism is associated with the risk of CRC. Although the rs3813867 and rs6413432 polymorphisms were not associated with the risk of CRC, subgroup analyses revealed some differences in the risk of association between Asians and Caucasians, and between high- and low-quality studies. Finally, in view of the limitations mentioned above, further studies with a better overall design are needed to verify the true association between *CYP2E1* polymorphisms and CRC risk.

## Methods

### Literature search strategy and study selection

A literature search was performed in PubMed, Web of Science and Scopus databases up to February 24, 2022. The following keywords were used: “CYP2E1” AND “polymorphism” AND “colorectal cancer”. No language restriction was set. Studies were included if they met the following criteria: (1) examined the association between *CYP2E1* gene polymorphisms and CRC risk; (2) case–control studies in design; and (3) contained sufficient data to estimate an odds ratio (OR) and its 95% confidence interval (CI). Non-research articles and studies conducted in non-human subjects were excluded. If more than one article was published by the same authors with the same or overlapping subjects, the study with the largest sample size or the most recent data was selected. References of eligible studies and relevant review articles were also screened to identify additional studies. The review was not prospectively registered.

### Data extraction

The following information was extracted from each included study: first author’s name, year of publication, ethnicity (categorized as Asian, Caucasian, or Africans), country, total number of cases and controls, allele and genotype frequencies, genotyping methods, and deviation from Hardy–Weinberg equilibrium (HWE). When the HWE *p*-value was not reported, it was calculated using a Pearson’s χ^2^ test. All extracted information was recorded in an Excel spreadsheet.

### Data synthesis

The quality of the included studies was assessed by two investigators using the Modified Newcastle–Ottawa Scale for Case–Control Studies of Genetic Association^64^. Studies that received ≥ 5 stars were considered to be of high quality. The strength of association between the *CYP2E1* polymorphisms and CRC risk was assessed using the odds ratio (OR) and the corresponding 95% confidence interval (CI). Statistical significance of the pooled ORs was determined using the Z-test, and a *P* < 0.05 was considered statistically significant. In addition, heterogeneity among the studies was assessed using Cochran’s *Q* statistic test and the *I*^2^ statistic to quantify the proportion of total variation due to heterogeneity. An *I*^2^ value ≥ 50% was considered as having significant statistical heterogeneity, for which a random-effects model (the DerSimonian–Laird method) was used to calculate the pooled OR. Meanwhile, when heterogeneity was low, the fixed-effects model (Mantel–Haenszel method) was used to calculate the pooled OR. Sensitivity analysis was also performed to assess the robustness of the results. In addition, subgroup analyses were performed according to the ethnicity of the participants and the methodological quality of the studies. To assess the presence of publication bias among the included studies, Begg’s funnel plot and Egger’s linear regression tests were performed. If publication bias was identified, a ‘trim and fill’ analysis was performed to detect missing studies. All analyses were performed using STATA software, version 14.0 (StataCorp LP, College Station, TX, USA).

## Supplementary Information

Supplementary Information.

## Data Availability

The datasets supporting the conclusion of this article are included within the article (and the online Supplementary Information file).
