# Supplementary material for: Association between CYP2E1 polymorphisms and colorectal cancer risk: a systematic review and meta-analysis
Source: Sci Rep. 2022 Nov 23;12:20149. doi: 10.1038/s41598-022-24398-w (PMC9684517; doi:10.1038/s41598-022-24398-w)
Supplement: Supplementary file 1 — Supplementary Information. [file 41598_2022_24398_MOESM1_ESM.docx]

**Supplementary Information**

**
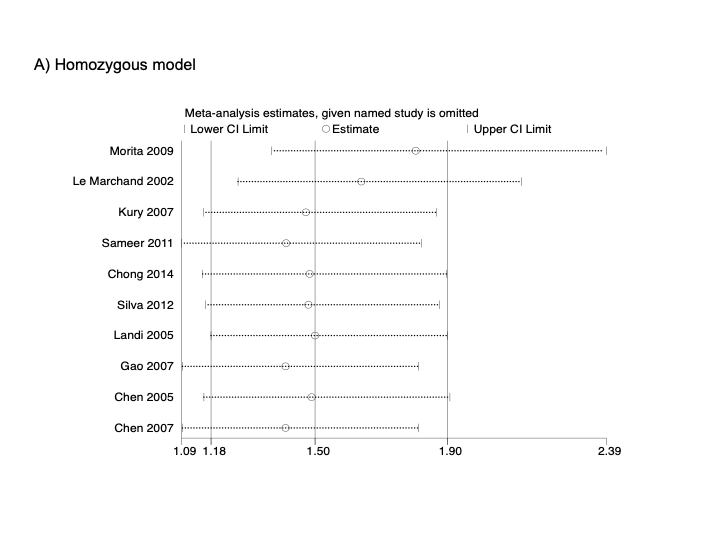
**

**
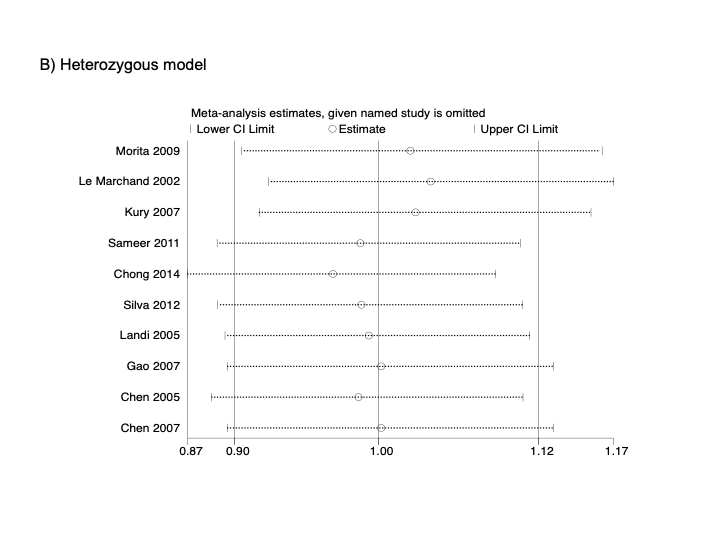
**

**
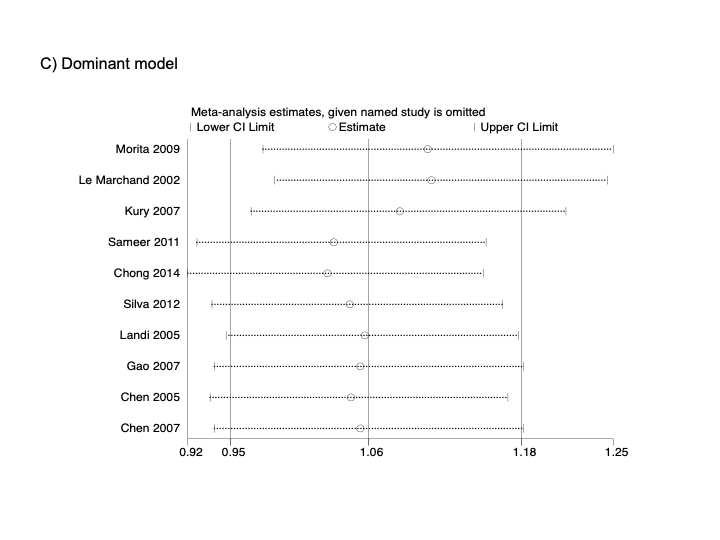
**

**
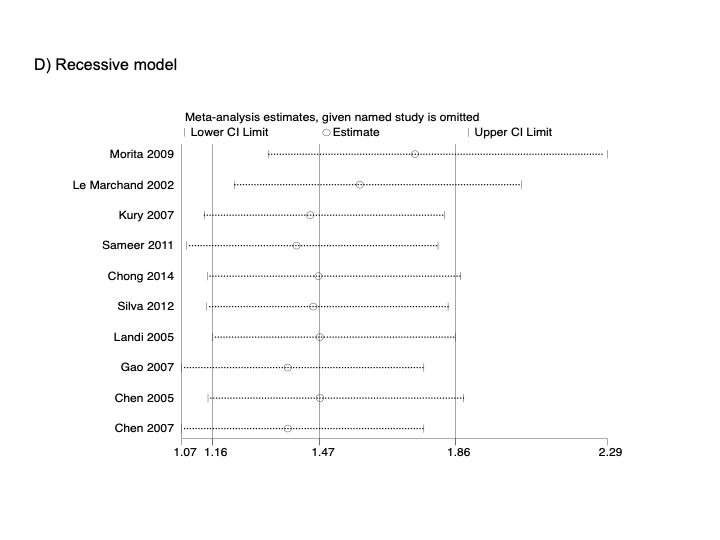
**

**
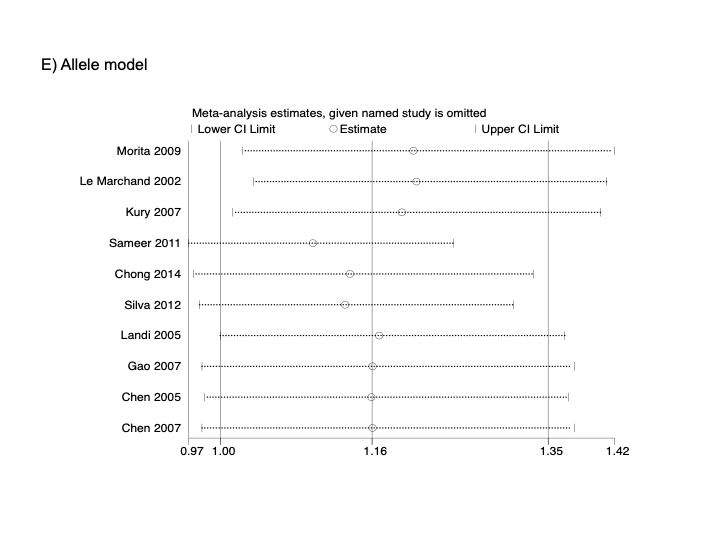
**

**Supplementary Figure S1.** Sensitivity analysis of *CYP2E1* rs2031920 and colorectal cancer risk


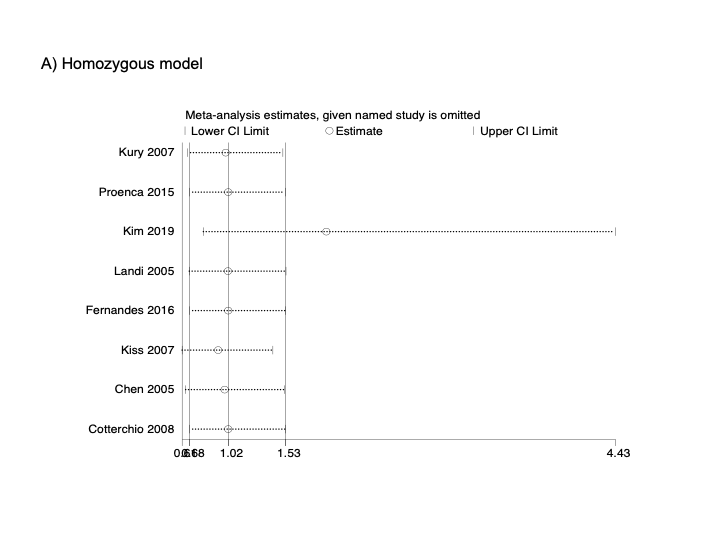


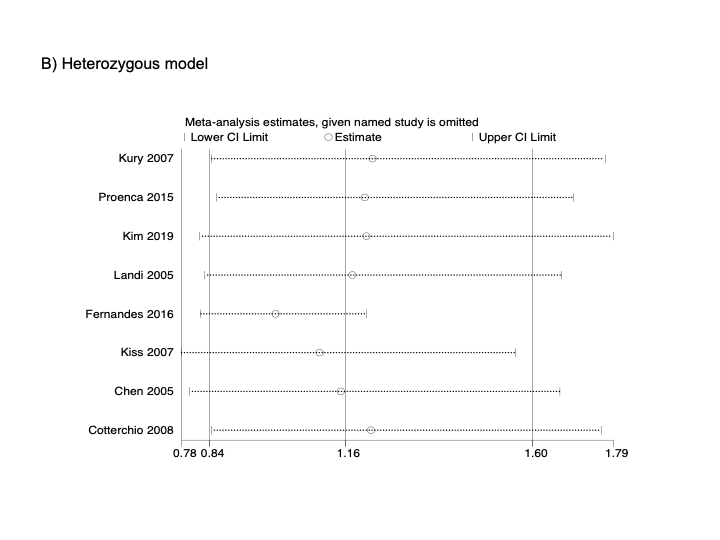


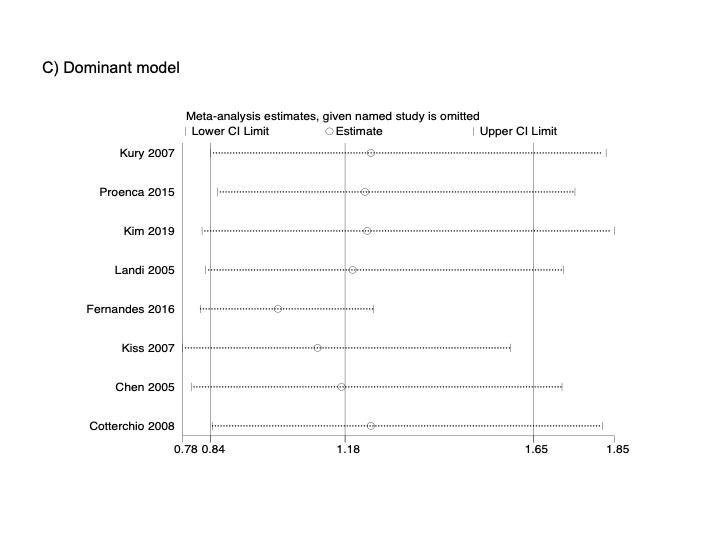


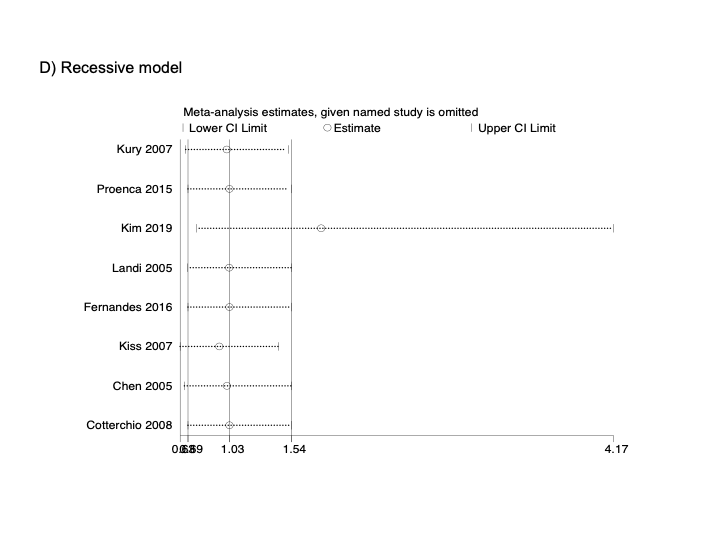


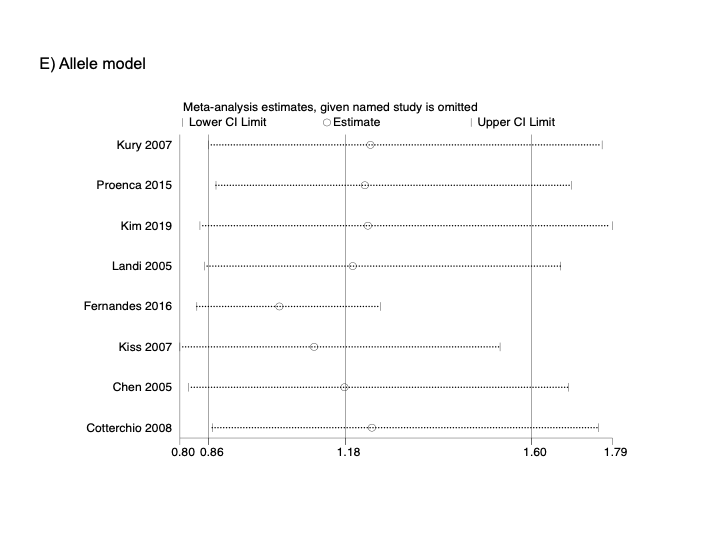
**Supplementary Figure S2.** Sensitivity analysis *CYP2E1* rs3813867 and colorectal cancer risk


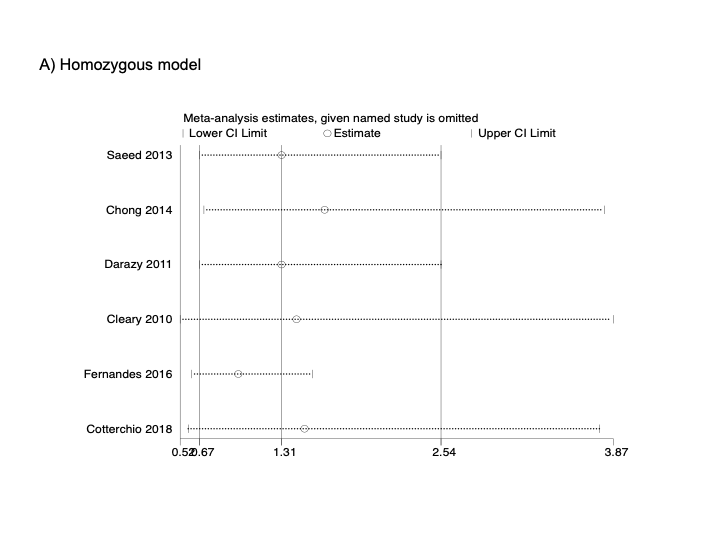


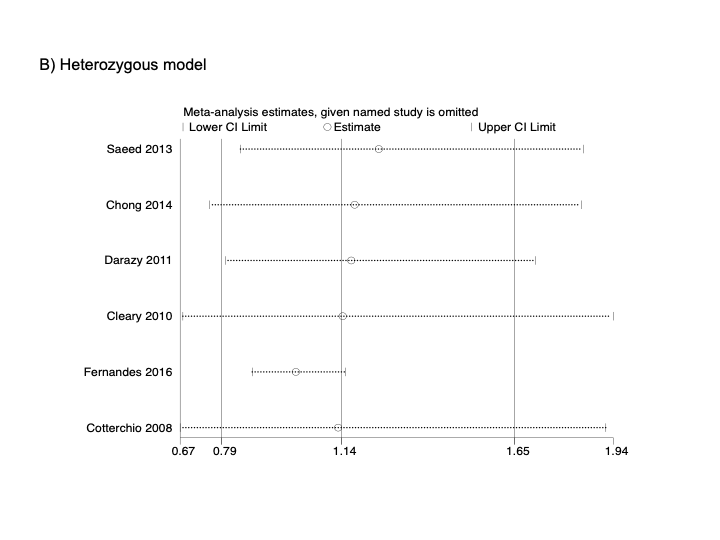


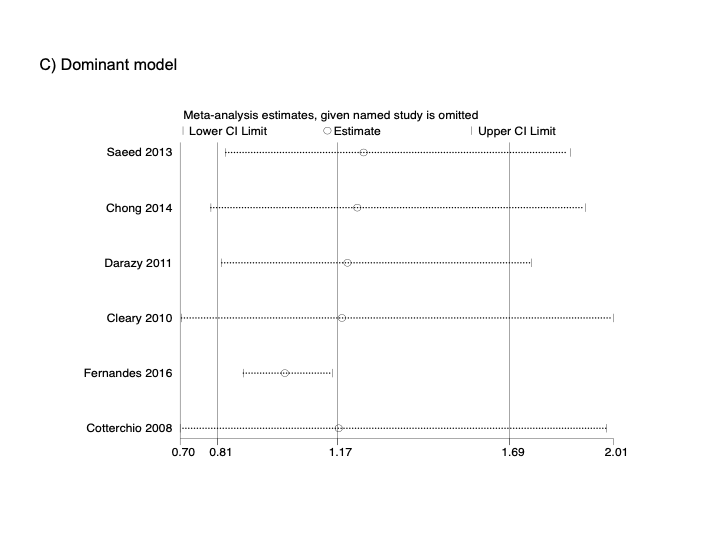


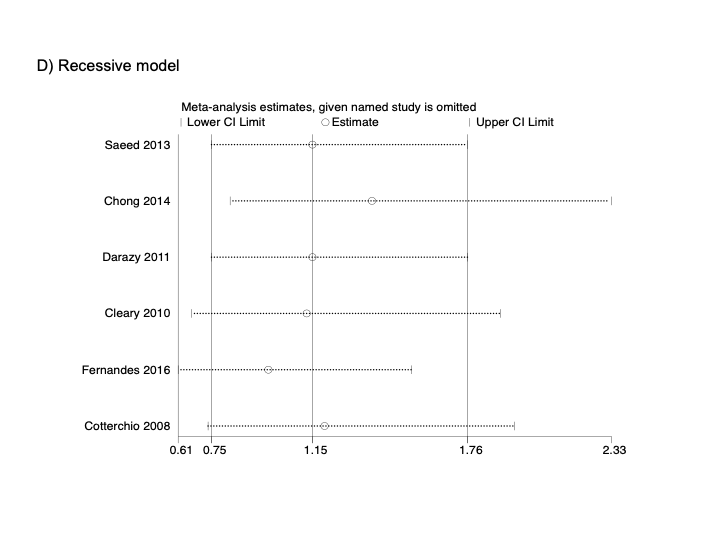


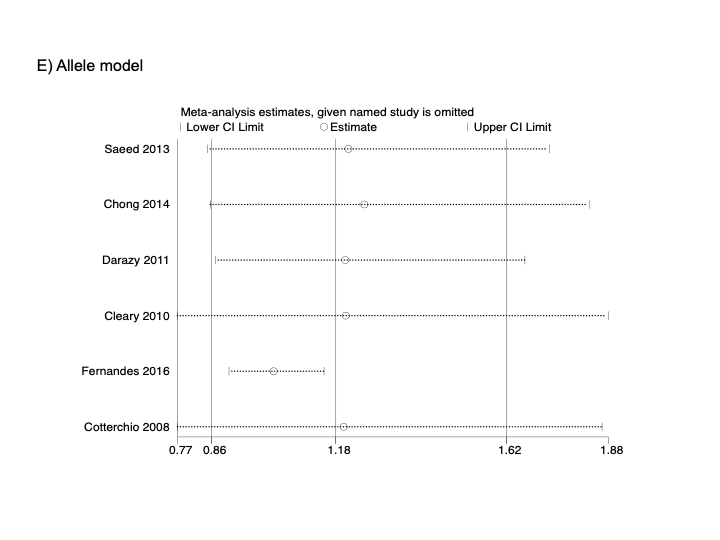
**Supplementary Figure S3.** Sensitivity analysis of *CYP2E1* rs6413432 and colorectal cancer risk
